# Unveiling the genetic blueprint of verbascoside biosynthesis in *Olea europaea*: an integrative metabolomics, genetics and transcriptomics approach

**DOI:** 10.1093/hr/uhag154

**Published:** 2026-04-17

**Authors:** Simone Pizzi, Roberto Mariotti, Sara Pinosio, Alice Cattivelli, Davide Tagliazucchi, Muhammad Ajmal Bashir, Lorenzo Cruciani, Soraya Mousavi

**Affiliations:** Institute of Biosciences and Bioresources, National Research Council, Perugia 06128, Italy; Institute of Biosciences and Bioresources, National Research Council, Perugia 06128, Italy; Institute of Biosciences and Bioresources, National Research Council, Sesto Fiorentino 50019, Italy; Nutritional Biochemistry Lab, Department of Life Sciences, University of Modena and Reggio Emilia, Reggio Emilia 42121, Italy; Nutritional Biochemistry Lab, Department of Life Sciences, University of Modena and Reggio Emilia, Reggio Emilia 42121, Italy; Institute of Biosciences and Bioresources, National Research Council, Perugia 06128, Italy; Institute of Biosciences and Bioresources, National Research Council, Perugia 06128, Italy; Institute of Biosciences and Bioresources, National Research Council, Perugia 06128, Italy

## Abstract

The olive tree (*Olea europaea* L.) is a key source of verbascoside, a potent phenylethanoid glycoside known for its antioxidant, anti-inflammatory, and neuroprotective properties. Traditional breeding for enhanced phenolic profiles is limited by the olive’s long juvenile period and complex genetics. This study dissected the unknown genetic architecture of verbascoside biosynthesis using a multi-omics strategy integrating metabolomics, Quantitative Trait Loci mapping, and transcriptomics across an F1 population and diverse cultivars. This approach identified a major genetic locus controlling verbascoside content on chromosome 21 (LOD [Logarithm of the Odds]  = 17), which explained the wide metabolomic variation. The locus includes 193 genes, of which 29 were involved in verbascoside synthesis and 47 showed differential expression. A homology-based analysis, using data from five high-verbascoside Lamiales species, narrowed the search to a small set of high-confidence candidate genes, including BAHD acyltransferases (BEAT, AHCT, HCBT, DAT acyltransferase family) and various cytochrome P450s (CYPs). Digital PCR confirmed the differential expression of nine candidate CYP genes. The expression of both *OeCYP71D55* (a premnaspirodiene oxygenase) and the precursor-producing *OeACy3G* peaked at the T5 stage, 165 days after flowering. This peak mirrored the maximum time of verbascoside accumulation, suggesting that the transcriptional regulation of these CYPs is a rate-limiting factor in the pathway. This study defines a critical genetic locus and a core set of candidate genes governing verbascoside production in olive cultivars. These discoveries provide valuable molecular tools to fast-track the breeding of new olive varieties with enhanced health benefits.

## Introduction

The olive tree (*Olea europaea* L.) holds economic importance through the production of olive oil and table olives. Beyond its oil, the olive fruits are rich in secondary metabolites [1–3]. Among the phenolic compounds in the olive, secoiridoids are derived from iridoids via a specific ring opening and subsequent modifications [4–6]. Oleuropein is abundant, constituting up to 14% of the olive fruit’s dry weight, while ligstroside has also been extensively studied for its role in chronic noncommunicable diseases [7, 8]. Phenolic alcohols, such as tyrosol and hydroxytyrosol can inhibit excessive reactive oxygen species production and exhibit higher radical-scavenging activity [9]. Flavonoids, primarily apigenin and luteolin, along with lignans, further contribute to the potent antioxidant capacity of olive phenols [5].

Verbascoside is a prominent phenylethanoid glycoside found in olives and numerous other medicinal plants, including *Echinacea* and *Plantago* species, as well as *Sesamum indicum* and *Rehmannia glutinosa* [10–12]. Extensive research has demonstrated its diverse and potent biological activities [4, 13–15]. During olive ripening, an inverse relationship between the concentrations of oleuropein and verbascoside is frequently observed. Oleuropein tends to decrease in mature drupes, while verbascoside accumulates, a trend that has been documented across numerous cultivars [16–18]. This negative correlation suggests a potential biochemical link, where the degradation of oleuropein may contribute to verbascoside synthesis. This hypothesis is supported by the fact that oleuropein breakdown can yield hydroxytyrosol, a component of the verbascoside molecule [19]. The biosynthesis of phenylethanoid glycosides, such as verbascoside, in *Olea europaea* involves pathways originating from L-phenylalanine and L-tyrosine [15]. Although recent studies in other plants, such as *Ligustrum robustum* and *Cistanche tubulosa* have successfully elucidated the complete biosynthetic pathways for verbascoside and related compounds [15, 20], these findings cannot fully clarify the process in olive. The ‘untapped biosynthetic potential’ for verbascoside, demonstrated in olive cell cultures [21], indicates that the genetic capacity for high-level production is present within the olive genome but may be constrained by negative regulation or precursor supply limitations in the whole plant.

Understanding the genetic and molecular mechanisms underlying phenolic biosynthesis in olives is crucial for developing cultivars with superior nutritional and sensory traits through breeding programs. Traditional breeding strategies face limitations due to the olive genome’s complex nature and the tree’s long generation time [22–24]. Advanced techniques, such as Quantitative Trait Loci (QTL) in olives, have been employed to identify genomic regions associated with various traits, including phenolic metabolite content [25–28]. Furthermore, RNA-Seq has revolutionized gene expression analysis, enabling the identification of genes and regulatory elements involved in complex metabolic pathways [1, 29]. Integrative approaches combining QTL and transcriptomic analyses have proven successful in deciphering the molecular mechanisms underlying secondary metabolism and quality traits in other plant species [30–32].

To bridge the gap between genetic potential and metabolic output, this study employed an integrative multi-omics strategy. First, QTL analysis of a bi-parental F1 population was used to identify the primary genomic regions controlling phenolic accumulation. Because QTLs often contain hundreds of candidate genes, we subsequently integrated transcriptomic data (RNA-Seq) across different developmental stages to refine the obtained output, filtering for functionally active differentially expressed genes (DEGs). Finally, whole genome sequencing (WGS) variant analysis was performed to determine if verbascoside quantity was driven by structural mutations in low and high content cultivars. Collectively, this rationale allowed us to pinpoint the specific candidate genes and rate-limiting steps governing verbascoside biosynthesis in *Olea europaea*.

## Results

### Phenolic compounds in F1 progeny

**Figure 1:**
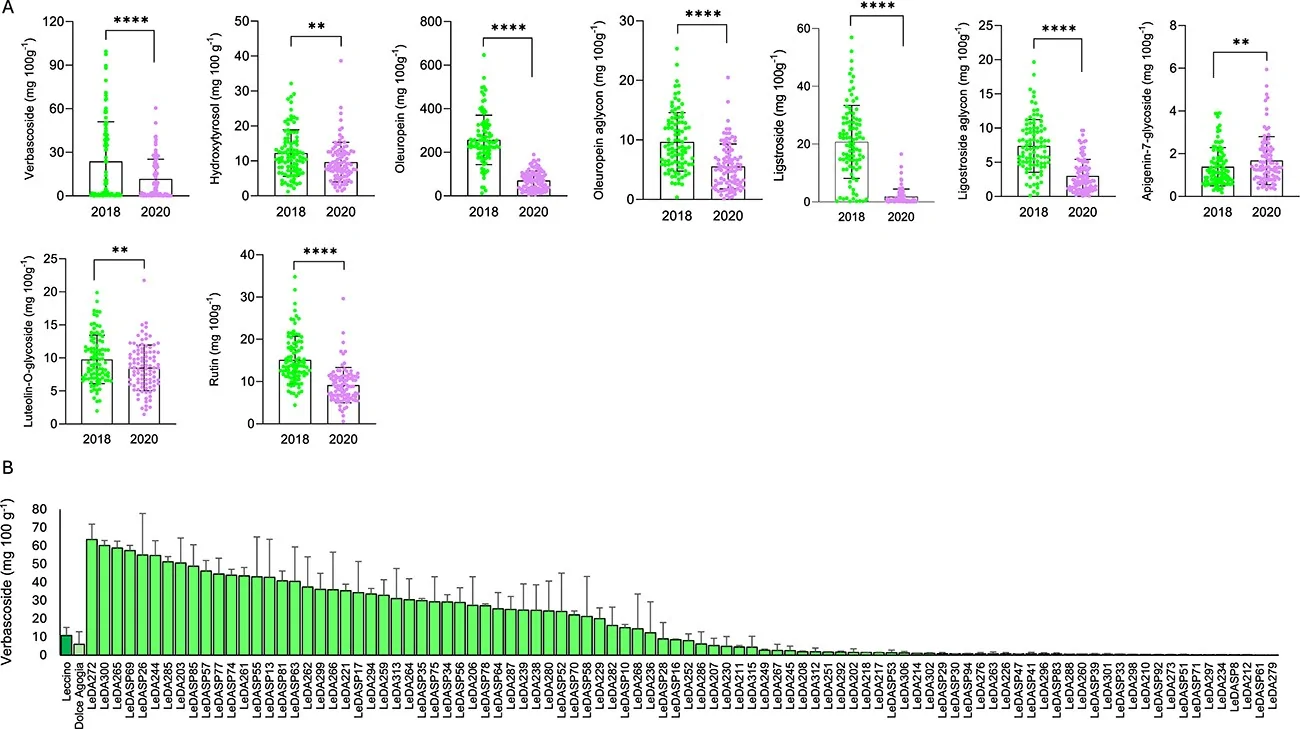
(A) Box and whiskers plots represent the distribution of the trait. These plots display a five-number summary of a set of data: the minimum and maximum, the first quartile, the median, and the third quartile, allowing a comprehensive data comparison. Tukey’s multiple comparisons test results show significant differences (^****^  *P* < 0.0001 and ^**^  *P* < 0.01) among phenotypic distribution. The circles represent 96 individuals. (B) Variation of verbascoside content in 96 individuals of the olive cross-population Leccino × Dolce Agogia. Histograms showing verbascoside content of olive fruits. Bars represent the means ± SD of three replicates. Content is expressed as mg 100 g^−1^ of fresh fruit pulp.

For all phenols analyzed, clear segregation was observed in the fruits of the cross population (Fig. 1A). Phenols’ concentration in the fresh fruit pulp showed significant variation; verbascoside, in particular, exhibited the highest variability, with levels ranging from 0.08 to 126 mg 100 g^−1^ across both years. The secoiridoid group also displayed wide variations. Oleuropein content ranged from 12.41 to 650 mg 100 g^−1^ in 2018 and from 5.5 to 190 mg 100 g^−1^ in 2020. Over the 2 years, the concentrations of other secoiridoids varied from 3.20 to almost 30 mg 100 g^−1^ for hydroxytyrosol, ~0.4 to 27 mg 100 g^−1^ for oleuropein aglycon. Ligstroside content changed from 0.18 to 31 mg 100 g^−1^ and finally, ligstroside aglycon ranged from 0.06 to 20 mg 100 g^−1^. Among the flavonoid compounds, the content variation over the 2-year study was 0.6 to 35 mg 100 g^−1^ for rutin, while luteolin-7-O-glucoside varied from 1.4 to 22 mg 100 g^−1^ and apigenin-7-O-glucoside from 0.18 to 6 mg 100 g^−1^.

The significant variability observed in fruit phenolic content highlights the importance of identifying the underlying genes and alleles, providing a foundation for using our genetic map to locate the genomic regions that control these levels.

### QTL analysis

QTL analysis was performed according to phenolic content and SNP data from the genetic map of the progeny [23]. QTLs associated with each phenolic compound were detected separately on each parental map. A total of nine significant QTLs for six different phenolic compounds were identified across six chromosomes (Chr4, Chr6, Chr7, Chr9, Chr13, and Chr21), both by using the stringent 5% genome-wide permutation threshold (LOD ≥ 3.39) and relaxed threshold (LOD ≥ 3) to capture broader interacting networks for subsequent transcriptomic filtering.

Four QTLs were mapped to the ‘Dolce Agogia’ genetic map, and five to the ‘Leccino’ map over the 2-year study period. The highest LOD score (17) was associated with verbascoside on the ‘Dolce Agogia’ parental map. This strong genetic signal reflects the wide variation in the verbascoside content within the progeny, accounting for 75% of phenotypic variation for this trait among progenies (Fig. 1B). A partial overlap of the 2-year QTLs was distinctly observed for verbascoside and apigenin-7-O-glucoside. Furthermore, the identified luteolin-7-O-glucoside QTL was found to be entirely included within the apigenin-7-glycoside QTL region (Table 1).

**Table 1:** QTLs for phenolic compounds in olive fruit pulp detected using the Leccino X Dolce Agogia maps.

**Phenolic compound**	**MAP**	**YEAR**	**LOD score**	**LG**	**QTL position in genetic maps**	**QTL position in reference genome**	**Total Mb**	**Genes**
**Oleuropein**	Dolce Agogia	2020	**3.4**	1	tig02154686:500000–1050000; tig00002150:45000–80000	Chr6:13292610–15201415	1.9	104
**Ligstroside**	Dolce Agogia	2018	3	13	tig00000950: 250000–1600000; tig00000470: 680000–2200000	Chr4:22765550–26634249	3.5	119
**Ligstroside aglycon**	Leccino	2018	**3.7**	18	tig00001996:45000–1700000; tig00008642:60000–70000; tig00000360:190000–210000	Chr9:9378590–12748501	3.6	123
		2018	3.2	8	tig00006525:33570–65792; tig00007914:47228–474628;	Chr13:44617793–46602643	2	53
**Verbascoside**	Dolce Agogia	2018	**17**	21	tig00003859:25000–470000 tig00001017:180000–730000 tig00008623:150000–600000 tig02154094:2500000–3000000	Chr21:23779043–27084393	3.3	158
		2020	**4.8**		**tig00001017:186358–1213798;** tig00003859:28889–465167; **tig00008623:140397–575032**; tig02154094:2580203–3042812;	Chr21:23662664–27084393	3.4	165
**Apigenin-7-O-glucoside**	Leccino	2018	**6.5**	6	tig00002243:1500000–1900000	Chr7:3752622–4814004	1	19
		2020	3		**tig00002243:163125–1511754**; **tig00004037:75481–659918**; **tig02155479:1661576–1765224**	Chr7:1468766–5066668	3.6	123

In bold the LOD scores indicate QTLs that exceed the strict 5% genome-wide significance threshold (3.39) determined by permutation tests.

QTL positions in bold are on the same chromosome but located in different places. Genes that fall in the QTL interval (according to the arbequina reference annotation) have been shown in the last column.

**Figure 2:**
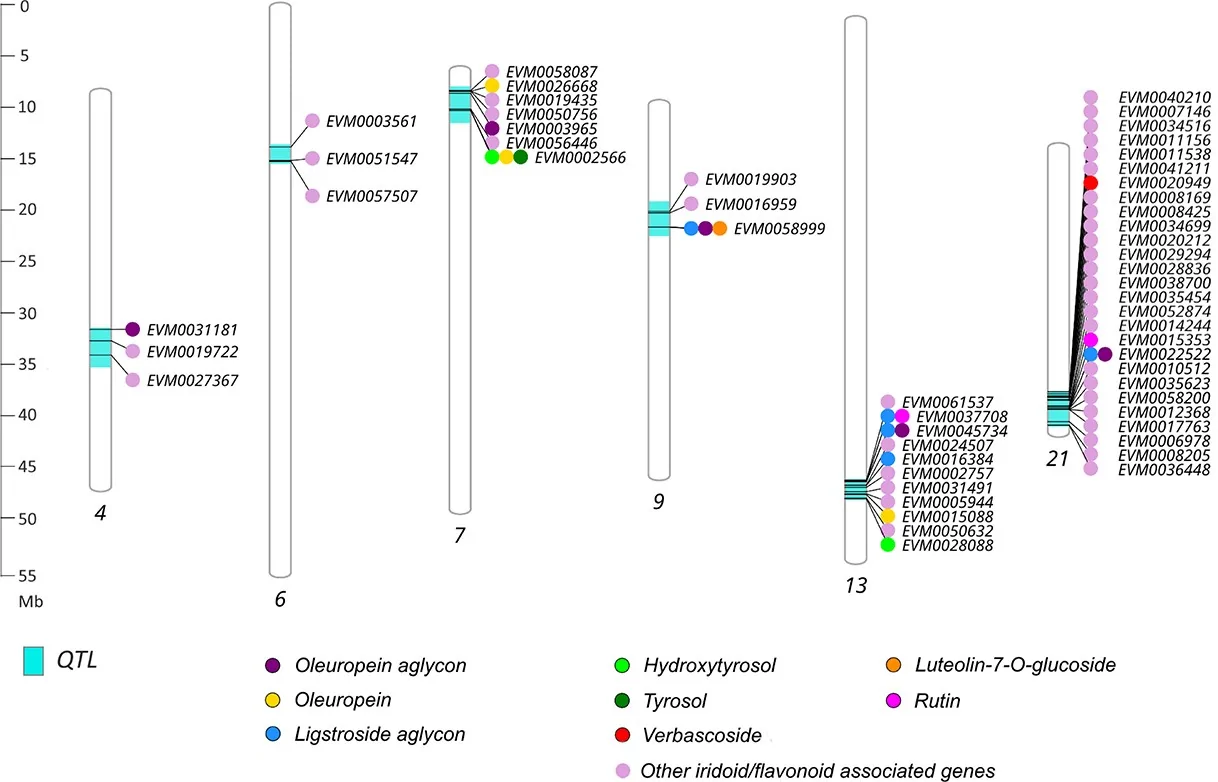
Ideogram of the six chromosomes of *Olea europaea* v. arbequina with the QTL region in cyan blue (LOD > 3). Dots indicate genes which are most likely involved in iridoid and flavonoid synthesis. Each color refers to the phenol for which a differential expression analysis was performed, except the light purple which indicates relevant genes non-differentially expressed, but that are well-known to be, or most likely are, involved in iridoid and flavonoid biosynthetic pathways. The light blue sections on the chromosomes denote the QTL regions. Individual colored dots represent genes identified through differential expression analysis for specific phenols. Specifically, dark purple indicates Oleuropein aglycon, yellow represents Oleuropein, and light blue corresponds to Ligstroside aglycon. Hydroxytyrosol and Tyrosol are marked by bright green and dark green dots, respectively. Red dots identify genes related to Verbascoside, orange dots represent Luteolin-7-O-glucoside, and pink dots signify Rutin. Finally, light purple dots are used for other iridoid or flavonoid associated genes.

Oleuropein aglycon, an important secoiridoid, had a score very close to the fixed threshold (LOD ≥ 3), namely >2.8 on the ‘Dolce Agogia’ map and >2.7 on the ‘Leccino’ map. We therefore included oleuropein aglycon in the analysis and extended the search to genes located within these QTL regions. Across all targeted QTL regions, a total of 780 genes were identified. The highest number of genes (193) was identified on Chr21, within the QTL region associated with verbascoside; of these 35 were related to transcription factors and zinc fingers. Among these, 29 genes were flagged as potentially involved in verbascoside and iridoid biosynthesis, based on previously published literature. These include genes encoding for enzymes such as polyphenol oxidase, cytochrome P450 CYP72A219 (a monooxygenase), anthocyanidin 3-O-glucosyltransferase 5 (a UDP-glycosyltransferase), and anthocyanidin 3-O-glucoside 6″-O-acyltransferase (an acyltransferase). Additionally, among the 119 genes identified in the QTL region for luteolin-7-O-glucoside and the 151 genes for apigenin-7-O-glucoside, 14 candidate genes were found that could be involved in flavonoid synthesis (Table S1 and Fig. 2). Finally, of the 146 genes mapped in the QTL regions for ligstroside aglycon, 14 were identified as potentially related to iridoid and flavonoid synthesis.

### Transcriptomic variations and shared pathways

**Figure 3:**
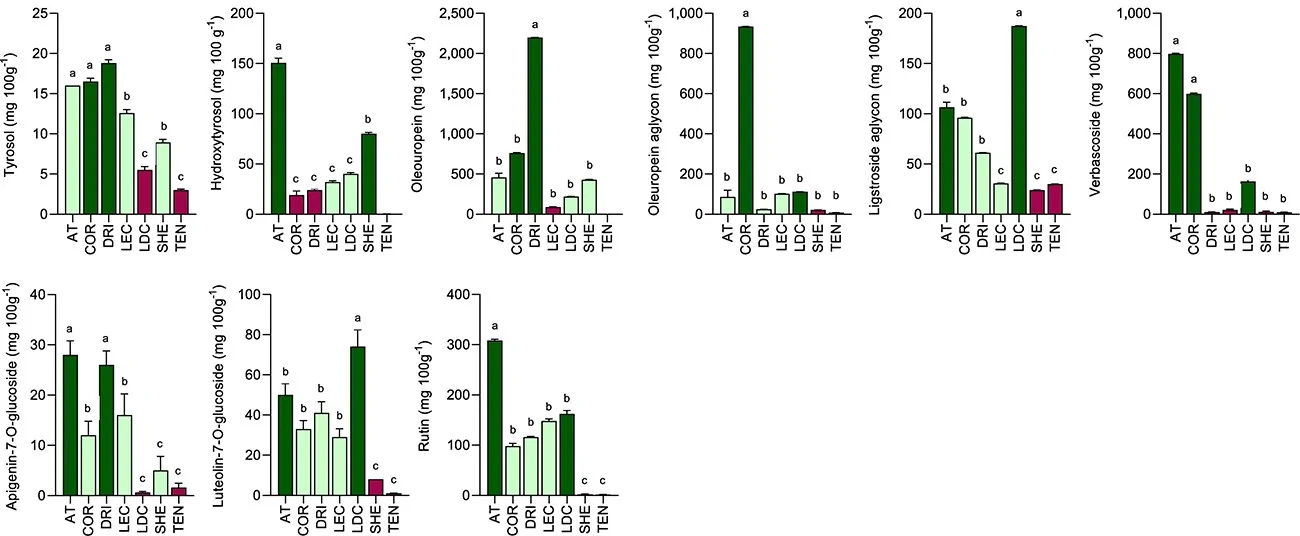
Variation of single phenolic compounds in seven selected cultivars for transcriptomic analysis. Bars represent the means ± SD of three replicates. Different letters correspond to significantly different values at *P* ≤ 0.05. Content is expressed as mg 100 g^−1^ of fresh fruit pulp. Dark green represents the highest concentrations, light green denotes medium concentrations, and dark red indicates low phenol concentrations.

To understand the transcriptional regulation of phenolic biosynthesis, we analyzed DEGs between cultivars with ‘High’ vs ‘Low’ content for nine distinct phenols at 165 days after flowering (DAF; Fig. 3). Using these categorizations, DEGs associated with each phenol were identified by contrasting the transcriptomes of the ‘High’ vs ‘Low’ groups across all five developmental time points (T1–T5). Genes found to be differentially expressed in at least one time point were compiled for each respective phenol.

**Figure 4:**
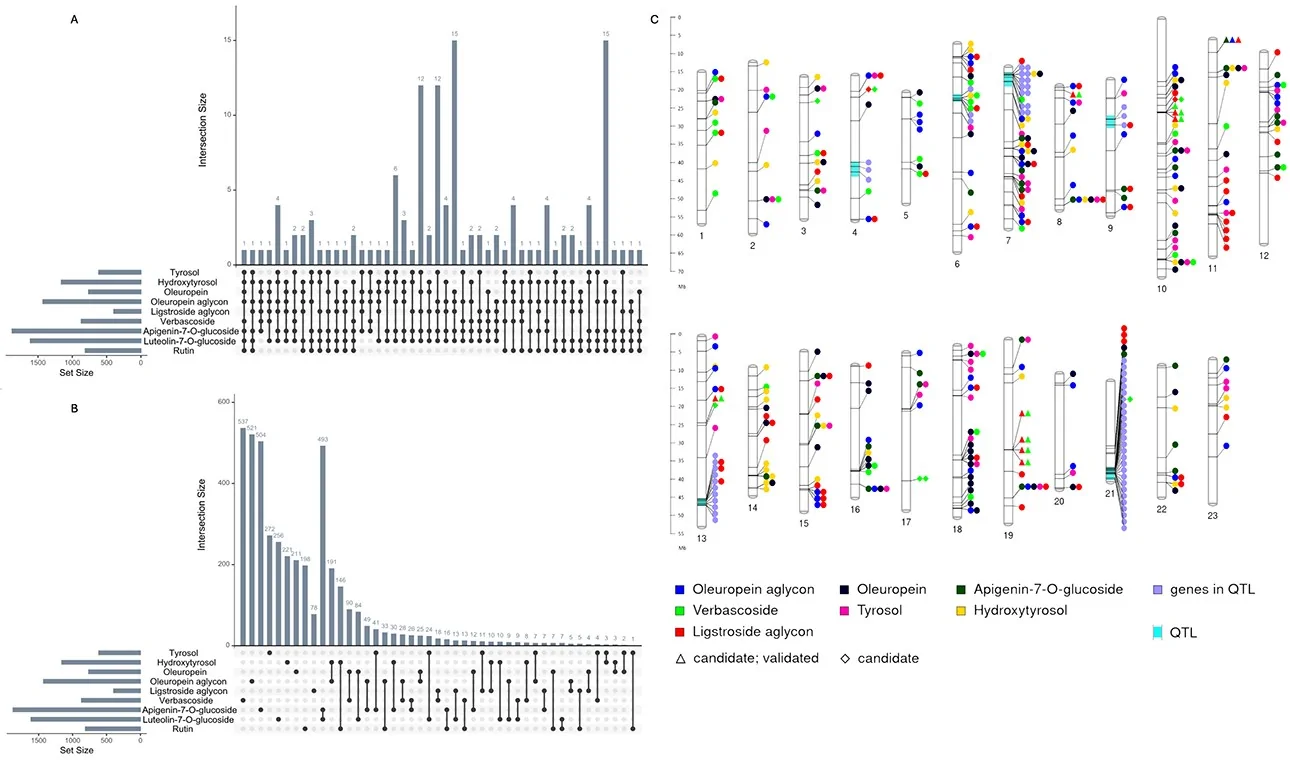
(A) Bar plots of the intersections among the 9 DEG sets. Shared genes among 5, 6, 7 and 8 phenol sets. (B) Number of unique genes for each phenol set, and genes shared between 2 phenol sets. (C) Ideogram of the 23 chromosomes of *Olea europaea* v. Arbequina. Regions highlighted in light blue represent QTLs identified on chromosomes 4, 6, 7, 9, 13 and 21. Dots indicate either loci of differentially expressed genes (DEGs), associated with six phenolic compounds, or genes within QTLs that are not DEG but are possibly involved in the phenol synthesis. Colors correspond to the specific phenol classes analyzed in the differential expression study (FDR < 0.05): dark purple for Oleuropein aglycon, yellow for Oleuropein, light blue for Ligstroside aglycon, bright green for Hydroxytyrosol, dark green for Tyrosol, red for Verbascoside, orange for Luteolin-7-O-glucoside, and pink for Rutin. Light purple indicates relevant genes non-differentially expressed, but that are well-known to be, or most likely are, involved in iridoid and flavonoid biosynthetic pathways. Triangle and rhombus indicate validated and candidate genes discussed in Table 2 and Table S24.

By taking the union of all the significant genes resulting from the nine separate differential expression (DE) analyses, a total number of 5344 unique DEGs were identified. Subsequently, the intersection of these nine sets was analyzed to reveal several shared genes acting across multiple phenolic pathways. Substantial overlaps were observed among the DEG sets; for instance, 198 DEGs were shared among apigenin-7-O-glucoside, oleuropein, and tyrosol. This high degree of shared genes suggests that multiple phenolic biosynthetic pathways rely on a tightly co-regulated genetic network rather than acting in isolation (Fig. 4A and B). DEGs likely involved in iridoid, verbascoside, and flavonoid synthesis were successfully mapped across all 23 chromosomes (Fig. 4C).

### Pathway enrichment by phenol class

To better understand the regulatory mechanisms, Gene Ontology (GO) and Kyoto Encyclopedia of Genes and Genomes (KEGG) enrichment analyses were evaluated across the specific phenol groups. *Tyrosol and Hydroxytyrosol*: analysis identified 1159 DEGs for tyrosol (Table S2, Fig. S1) and 869 for hydroxytyrosol (Table S3, Fig. S2). Down-regulated tyrosol DEGs showed significant enrichment in secondary and phenylpropanoid metabolism (*P* < 0.01), as well as heme binding and monooxygenase activity. Hydroxytyrosol DEGs were associated with steroid and isoprenoid metabolic processes, alongside isomerase and UDP-glycosyltransferase activities. *Oleuropein and Ligstroside Derivatives*: we identified 1428 DEGs for oleuropein, 1876 for oleuropein aglycon, and 1611 for ligstroside aglycon (Tables S4–S6; Figs S3–S5). Consistently across these three phenotypes, down-regulated genes were heavily enriched in secondary metabolite, phenylpropanoid, and coumarin metabolic/biosynthetic processes. *Verbascoside*: among the 615 DEGs identified (Table S7), up-regulated genes were primarily driven by ligase activity and involved in phenylpropanoid and coumarin metabolic pathways (Fig. S6). *Flavonoids (Apigenin-7-O-glucoside, Luteolin-7-O-glucoside, Rutin)*: these phenotypes yielded 811, 395, and 763 DEGs, respectively (Tables S8–S10; Figs S7–S9). While apigenin and luteolin DEGs featured varied secondary metabolism and antioxidant activities, rutin up-regulated DEGs were notably enriched for glucosyltransferase activity (*P* < 0.02), directly relating to flavonoid synthesis.

To summarize, GO terms for biological processes and molecular functions indicated that the majority of down-regulated DEGs across all nine phenols were involved in phenylpropanoid and secondary metabolite biosynthesis. Furthermore, the specific enrichment of coumarin biosynthetic processes—alongside monooxygenase, ligase, transferase, and oxidase activities—among the DEGs for oleuropein, oleuropein aglycon, ligstroside aglycon, and verbascoside provides strong evidence of their direct regulatory role in iridoid synthesis.

### QTLs and DEGs data integrated analysis

By intersecting the QTL regions with our DEG datasets, we identified a total of 91 DEGs physically located within the QTLs (Tables S11–S19). The Chr21 verbascoside QTL contained the highest number of these mapped DEGs (47 genes). Crucially, this included three genes from the cytochrome P450 CYP72 family, notably 7-deoxyloganic acid hydroxylase (*7DLH*, EVM0022522), which was consistently up-regulated during fruit development in high-iridoid cultivars (Fig. S10).

Beyond verbascoside, mapped DEGs within other phenolic QTLs revealed broad functional overlaps. For instance, the oleuropein and luteolin-7-O-glucoside QTLs contained overlapping DEGs enriched for flavonoid biosynthesis (e.g. chalcone synthase) and UDP-glycosyltransferase functions. Similarly, a beta-galactosidase gene, known to catalyze the cleavage of β-glycosidic bonds, was commonly differentially expressed across the oleuropein aglycon, ligstroside aglycon, and luteolin-7-O-glucoside networks (Tables S14, S15, and  S18). Together, this spatial mapping highlights a complex, shared regulatory machinery governing multiple phenolic branches.

### Candidate genes identification for the synthesis of verbascoside

Of all nine phenolic compounds studied, verbascoside exhibited the highest phenotypic variability, with its concentration ranging from 0.08 to 126 mg 100 g^−1^. This extreme range of variation observed, together with the optimal segregation in the progeny, provides a valuable phenotypic signal, making it an ideal target to identify the genetic architecture that controls its synthesis. The LOD score of 17 is significantly higher than the threshold for significance (LOD ≥ 3) and any other reported in the study, pointing to a major genetic locus controlling its synthesis. The QTL region on Chr21 contained 193 genes; and for 29 of them, the annotations indicate a clear association to the verbascoside synthesis, the highest number of genes reported for any compound. The enrichment of these QTL regions with DEGs further solidified this focus, as the verbascoside QTL regions contained the highest number of DEGs (47 genes). Crucially, within this group, specific and highly relevant candidate genes were identified, such as three genes from the cytochrome P450 CYP72 family, which are known to be involved in iridoid biosynthesis.

Based on these findings, the biosynthetic pathway of verbascoside [15] was deeply analyzed. The most significant DEGs and QTL genes involved in iridoid and verbascoside synthesis (301 in total) were selected for an additional GO and KEGG pathway enrichment analysis, and the result was analyzed focusing on tyrosine metabolism and phenylpropanoid biosynthesis. As expected, most of the selected DEGs were involved in secondary metabolite, phenylpropanoid, and coumarin biosynthesis, with molecular functions related to heme binding, monooxygenase activity, and UDP-glycosyltransferase activity (Fig. S11). The KEGG pathway enrichment analysis identified 26 enzymes associated with phenylpropanoid biosynthesis, which revealed the pathway from L-phenylalanine to caffeic acid. Upstream in this pathway, the analysis identified tyrosine ammonia-lyase (TAL; [EC:4.3.1.24]), the enzyme that converts tyrosine to p-coumaric acid. Furthermore, 4-coumarate-CoA ligase-like 9 (4CL9; [EC:6.2.1.12]), which produces p-coumaroyl-CoA, was also identified in the enriched pathway (Fig. S12).

BAHD acyltransferases [**B**EAT (Benzylalcohol O-acetyltransferase), **A**HCT (Anthocyanin O-hydroxycinnamoyltransferase), **H**CBT (Anthranilate N-hydroxycinnamoyl/benzoyltransferase), **D**AT (Deacetylvindoline 4-O-acetyltransferase)], the primary enzymes in verbascoside synthesis, were identified in the phenylpropanoid pathway (Fig. S12). One of these, spermidine hydroxycinnamoyl transferase (SHT; EC 2.3.1.133), was previously shown to catalyze the regioselective acylation of salidroside and *p*-Coumaroyl-CoA to form osmanthuside A. Additionally, a rhamnosyl transferase involved in the conversion of osmanthuside A to osmanthuside B was identified in the pathway, a role potentially filled by anthocyanidin 3-O-glucoside 2-O-glucosyltransferase (ACy3G; [EC 2.4.1.111]). The final enzyme in the pathway, CYP98 hydroxylase (cytochrome P450 98A3; [EC 1.14.14.96]), was also identified. This enzyme was previously shown to complete the biosynthesis of verbascoside by catalyzing the *meta*-hydroxylations of the *p*-coumaroyl and tyrosol moieties of osmanthuside B.

**Figure 5:**
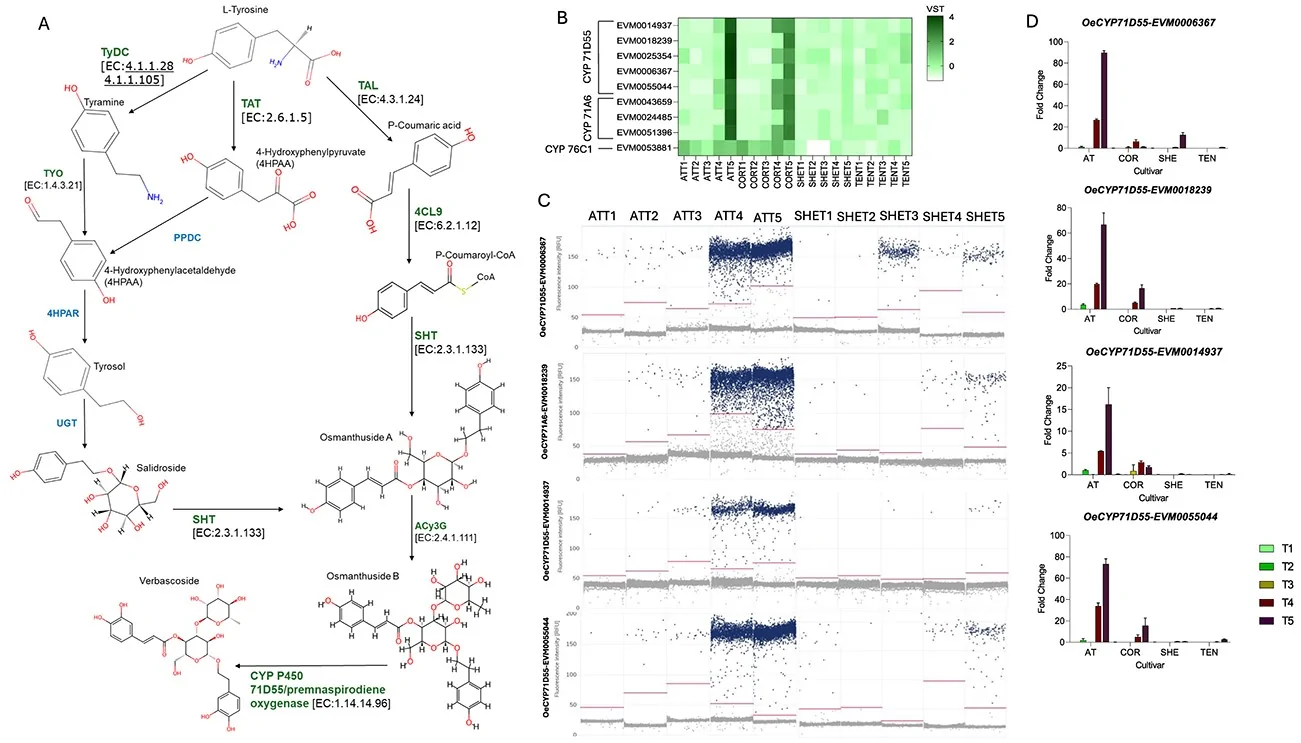
(A) Presumed verbascoside biosynthetic pathway in olive based on the KEGG pathways of phenylpropanoid and tyrosine metabolisms [15, 20]. The enzymes identified in KEGG maps and verified through transcriptomic analysis are shown in green, and enzymes verified only in transcriptomic analysis are shown in blue. (B) Heatmap obtained from nine distinct DEGs of the CYP in cultivars with high verbascoside content (‘Ascolana Tenera’ and ‘Coratina’) vs those with low content (‘Shengeh’ and ‘Tendellone’); the VST in positive (dark green) and negative (light green). (C) Scatterplots show the absolute quantification of an amplified transcript in 8.5 k 96-well of the digital PCR. Graphs of the measurements for the fluorescence signal (amplitude indicated on the y-axis) emitted by the amplification of specific fragments for the selected genes. (D) Relative quantification by dPCR of the mRNA expression of four genes based on the absolute concentration of the target genes which was then normalized using the reference gene (EF1α). Tukey’s multiple comparisons test was performed between each cultivar for each time point. The results show significant differences for the last two time points (4 and 5) (^****^P < 0.0001) and between high and low verbascoside content cultivars in all reported genes. The colors correspond to five different developmental stages or time points: light green for T1, bright green for T2, olive green for T3, dark red for T4, and dark purple for T5.

Furthermore, KEGG pathway mapping revealed that 12 key enzymes of our DEGs and QTL genes set are involved in upstream tyrosine metabolism (Fig. S13). This includes the alternative biosynthetic route from tyrosine to tyrosol or salidroside. Core upstream enzymes for these reactions, such as tyrosine decarboxylase (TyDC), tyramine oxidase (TYO), and tyrosine aminotransferase (TAT), were robustly identified in our pathway analysis. While DEGs for downstream steps like 4-hydroxyphenylacetaldehyde reductase (4HPAR) and UDP-glucosyltransferase (UGT) were also present, they lacked broader pathway enrichment. Based on these integrated results, we reconstructed a comprehensive, putative verbascoside biosynthetic pathway in olive (Fig. 5A), consistent with models from other high-producing Lamiales species.

To pinpoint specific enzymes within the olive verbascoside pathway, we performed a targeted homology analysis using 13 verified enzymes from five high-verbascoside Lamiales species (Table S20). By aligning these sequences against the Arbequina proteome and filtering for high-confidence matches (≥25% identity), we identified 249 putative olive homologs. Cross-referencing these homologs with our previously identified DEGs highlighted a subset of genes exhibiting significant expression variations during fruit development. Because cytochrome P450s are rate-limiting drivers of this pathway, we further refined 87 olive CYP homologs by comparing them with well-characterized enzymes from *Ligustrum robustum* and *Cistanche tubulosa*. This integrative filtering successfully isolated 15 high-confidence olive candidate enzymes (Table 2).

**Table 2:** Homology analysis for the main candidate enzymes p-coumarate-3-hydroxylase from *Ligustrum robustum* (Lr) and cytochrome P450 CYP98A from *Cistanche tubulosa* (Ct) which are responsible for verbascoside synthesis vs Arbequina genome (blastx for Lr; blastp for Ct).

**GeneID**	**Description**	**Identity with Lr (%)**	**Identity with Ct (%)**	**e-value with Lr**	**e-value with Ct**	**Bit score with Lr**	**Bit score with Ct**	**VST**
**EVM0051396**	Cytochrome P450 71A6-like	34.524	32.857	1,82E-85	9.90e-80	270	255	3.52
**EVM0039155**	Trans-cinnamate 4-monooxygenase	33.643	34.965	2 0e-76	1.07e-74	247	243	−1.27
**EVM0023651**	Cytochrome P450 71A6-like	33.615	33.200	8,80E-94	1.75e-99	294	309	2.52
**EVM0018239**	Premnaspirodiene oxygenase-like	32.993	33.555	8,69E-54	1.57e-51	184	177	3.72
**EVM0022465**	Ferruginol synthase-like	32.903	.	3,89E-85	7.37e-82	271	263	2.70
**EVM0053881**	Cytochrome P450 76C1-like	32.022	29.867	1 7e-42	1.73e-36	157	140	3.81
**EVM0050359**	Geraniol 8-hydroxylase-like	31.906	35.098	8,39E-88	1.61e-95	278	298	−1.33
**EVM0055044**	Premnaspirodiene oxygenase-like	31.510	.	2,36E-78	2.95e-80	254	259	3.71
**EVM0024485**	Cytochrome P450 71A6-like	31.210	31.423	1 9e-77	2.64e-82	252	264	3.21
**EVM0043659**	Cytochrome P450 71A6-like	31.153	29.394	4,98E-46	4.70e-48	165	170	4.13
**EVM0054016**	Premnaspirodiene oxygenase-like	30.846	.	6,04E-55	5.79e-62	191	210	2.46
**EVM0006367**	Premnaspirodiene oxygenase-like	30.804	.	2,24E-71	6.31e-74	236	243	3.49
**EVM0014937**	Premnaspirodiene oxygenase-like	30.599	.	5,88E-71	4.87e-80	235	259	3.37
**EVM0025354**	Premnaspirodiene oxygenase-like	30.599	30.392	2,92E-73	2.97e-73	241	241	3.08
**EVM0020949**	Cytochrome P450 CYP72A219-like	24.737	28.889	6.72e-10	4.85e-08	61.2	55.1	−1.72

The only CYP homolog in both our QTL region of verbascoside and the list of 249 DEGs was a cytochrome P450 CYP72A219-like enzyme, whose gene was down-regulated (−1.72 LFC) in the high verbascoside cultivars. Nine enzymes showed differential expressions between the cultivar with high vs the low verbascoside content (Fig. 5B) with positive variance stabilization transformation (VST) >3.

To validate the involvement of candidate genes in verbascoside synthesis, the expression of nine cytochrome P450 (CYP) genes and one anthocyanidin 3-O-glucoside 2 (*OeACy3G*) were quantified using digital PCR (dPCR; Fig. S14). Absolute transcript copy number analysis confirmed the transcriptomic findings (Fig. 5C) by revealing significant differences within cultivars across developmental stages (T1–T5) and between cultivars with high vs low verbascoside content. Relative expression analysis showed significant differences (*P* < 0.0001) for all analyzed CYP genes when comparing high and low verbascoside cultivars; almost all studied CYP genes displayed their highest expression at the T5 stage (Fig. 5D). Conversely, expression was nearly zero at 165 DAF in low-verbascoside cultivars, confirming the direct and essential role of these CYP genes in the synthesis pathway. Furthermore, the *OeACy3G* gene, which produces the precursor osmanthuside B from osmanthuside A, showed its highest expression starting at T4 and persisting into T5 (Fig. S14), supporting its role in the final stages of fruit development and verbascoside synthesis.

### Structural variants and polymorphisms in olive WGS dataset

**Figure 6:**
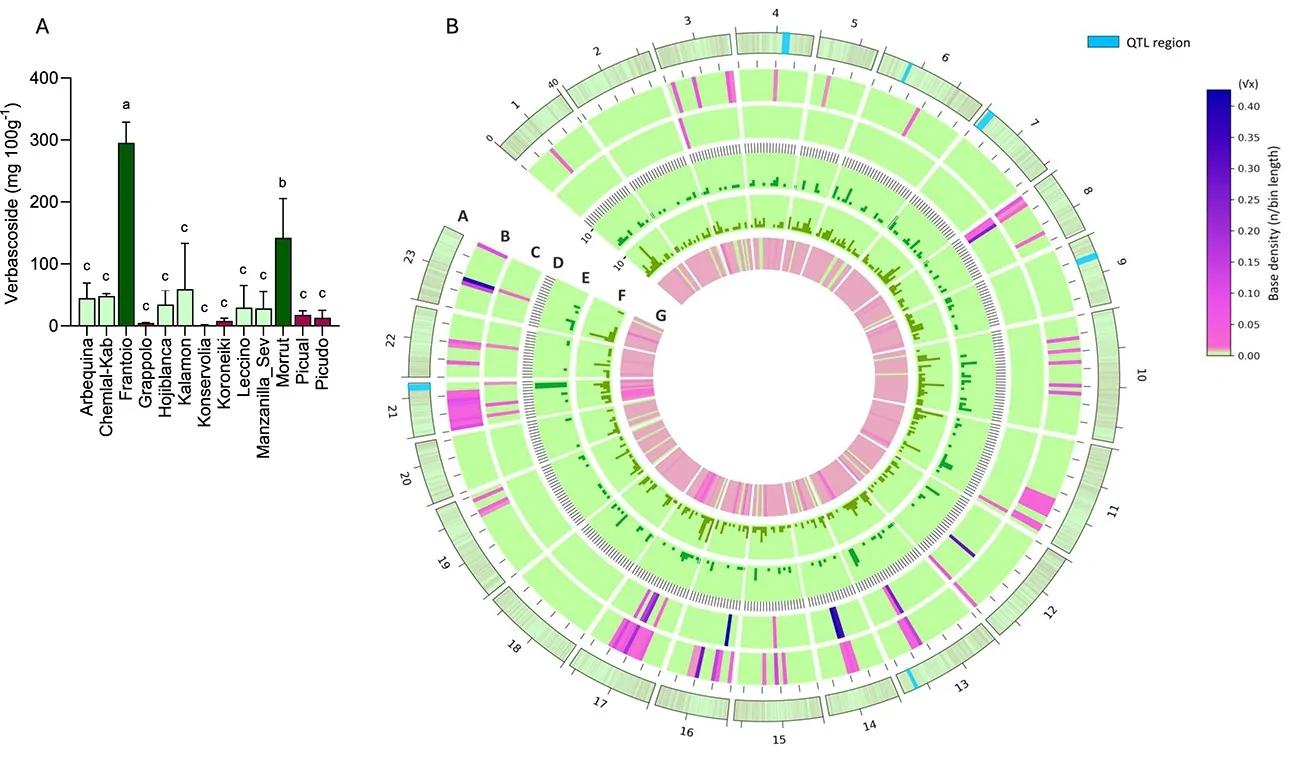
(A) Variation of verbascoside content in 13 selected cultivars for structure and SNP variation analysis. Bars represent means ± SD of three replicates. Different letters correspond to significantly different values at *P* ≤ 0.05. Content is expressed as mg 100 g^−1^ of fresh fruit pulp. Dark green represents the highest concentrations, light green denotes medium concentrations, and dark red indicates low phenol concentrations. (B) Circos plot shows exclusively variants present in Frantoio and Morrut, and simultaneously absent in Grappolo, Konservolia, Koroneiki, Picual, Picudo. Light blue represent QTLs identified on chromosomes 4, 6, 7, 9, 13 and 21. (A) Chromosomes, with thin bands representing genes, (B) deletions >100 bp, (C) insertions, inversions, duplications >100 bp (D) bins of 2.265 Mb, (E) genes important for verbascoside synthesis, (F) DEGs (G) SNPs, INDELs <100 bp. In B, C, and G, the color intensity reflects the density of bases. A small constant (10^–4.11^) was added to density values (root-square transformed). The bar height in tracks E and F indicates the number of genes within each bin.

To explore the polymorphisms within the selected candidate homologs in Arbequina related to the verbascoside synthesis, we searched for available, previously published, genomes of the 13 cultivars for which we had phenotypic data (Fig. 6A). As for differential expression, the comparison in this variants analysis was made between cultivars with high verbascoside content (Frantoio, Morrut) and those with low content (Grappolo, Konservolia, Manzanilla de Sevilla, Picual, Picudo). The circos plot (Fig. 6B) gave a comprehensive overview of the genomic distribution and abundance of variants found in these two ‘High’ cultivars but missing in the five ‘Low’ cultivars.

All structural variants identified in the Frantoio and Morrut cultivars were heterozygous. We did not observe a clear correlation between the size or position of these structural variants and their location within a QTL region. The only exception was a short, 13-bp inversion within verbascoside QTL, which did not affect any genes. A 766-bp deletion was detected 1.8 Mb upstream of the verbascoside QTL, affecting a glucosidase enzyme not considered to be associated with phenylpropanoid synthesis (Table S20). Among DEGs, eleven were affected by structural variants. For instance, the DEG on chromosome 14 encoding for UDP-glycosyltransferase 90A1-like was partially involved in a large duplication event. Polymorphisms, such as both SNPs and small INDELs (<100 bp), were assessed by identifying a total of 3668 variants in coding sequences.

For this study, 356 genes of previously discussed homologs and DEGs, as well as other genes potentially associated with verbascoside synthesis, have been analyzed (Table S21). A small subset of 16 genes contained variants with a predicted functional impact; and among these, there were four of the analyzed homologous DEGs: EVM0046379, EVM0013524, EVM0040812 and EVM0032848 (Table S22); a polyphenol oxidase homolog, with seven missense variants; a UDP-glycosyltransferase 83A1-like homolog, with six missense and two nonsense variants; a shikimate O-hydroxycinnamoyltransferase-like homolog, with three missense variants; phenylalanine ammonia-lyase, cytochrome P450 71A6-like (EVM0032848) and anthocyanidin 3-O-glucoside 6″-O-acyltransferase, each with two missense variants. Three missense variants in premnaspirodiene oxygenase gene have been found, located within the oleuropein QTL region on chromosome 6 (Table S23).

The low number of variants with a predicted functional impact suggests a high degree of conservation of the verbascoside pathway gene sequences. However, the identified specific mutations in conserved regions could still significantly impact their level of expression or protein function. The results strongly suggest that high-verbascoside olive cultivars (Frantoio, Morrut) express more effective isoforms of the enzymes needed to produce verbascoside. In contrast, low-verbascoside cultivars are likely to have isoenzymes that are less efficient or non-functional due to these specific missense and nonsense mutations.

## Discussion

Traditional breeding of olive trees is very slow and challenging due to the species’ long juvenility and complex genetic background [23, 24]. This research highlights the power of modern genomic tools to accelerate genetic gain. While QTL mapping has previously been used to identify genomic regions associated with olive phenols [25, 27], this study advances the field by layering transcriptomic data onto a high-confidence QTL. This integrative strategy, successful in other complex crops like grapevine, apple and peach [30–32], allows for the refinement of candidate gene lists. By correlating gene expression patterns with phenotypic variation across different cultivars and developmental stages, we were able to distinguish genes that are merely co-located within QTL from those that are functionally active and relevant to the trait of interest. The goal of this approach was to pinpoint the genetic components governing the rate-limiting steps in verbascoside biosynthesis.

Multi-cultivar DEG mapping and WGS analysis establish the Chr21 Dolce Agogia QTL as a central, highly conserved locus controlling verbascoside biosynthesis across different cultivars. The remarkable LOD score of 17, explaining 75% of the phenotypic variation, points to a single locus or a cluster of tightly linked genes acting as the primary genetic determinant for verbascoside levels in this population. This genetic signal corresponds with the vast range of verbascoside concentrations observed in the progeny, making this locus an ideal target for molecular dissection. The accumulation of verbascoside, like that of most secondary metabolites, is a classic quantitative trait, governed by the interplay of multiple genes and subject to significant environmental influence [33, 34]. This architecture is consistent with findings in other perennial fruit crops: studies in apple (*Malus domestica*), for instance, have revealed QTL hotspots on several linkage groups (LG1, LG8, LG13, LG16) that regulate entire classes of phenylpropanoids [35], while in Asian plum (*Prunus salicina*), multiple QTLs control anthocyanin content [36].

Overlapping DEGs highlight shared regulatory machinery among compounds with common biosynthetic origins. For instance, the massive overlap of 198 DEGs linking apigenin-7-O-glucoside, oleuropein, and tyrosol reflects a tightly co-regulated network, consistent with tyrosols’ role as an oleuropein precursor and their shared phenylpropanoid origins [16, 17]. For these and related phenols, enriched GO terms consistently implicate secondary metabolite pathways, specifically highlighting monooxygenase (cytochrome P450s) and ligase (e.g. 4-coumarate-CoA ligase) activities. Notably, these pathways are down-regulated at 165 DAF in high-phenol cultivars, suggesting bulk synthesis is complete and feedback inhibition has initiated [37].

The integration of RNA-Seq data was critical in narrowing down the 193 genes within the verbascoside QTL to a manageable list of functionally relevant candidates. The identification of 47 DEGs—including key enzymatic families like CYPs, acyltransferases, and UDP-glycosyltransferases—within the Chr21 QTL region provides strong evidence for its role in the verbascoside biosynthetic pathway. An interesting finding is the down-regulation of CYP72A219 in high-verbascoside cultivars. The CYP72 family member identified in our results, 7-deoxyloganic acid hydroxylase (7DLH), also located within the QTL, suggests a possible biochemical or regulatory link between verbascoside and iridoid/secoiridoid biosynthesis (e.g. oleuropein and ligstroside aglycon). Interestingly, while we identified a CYP72 family member annotated as 7DLH (EVM0022522) within this QTL, a recent functional characterization [38] revealed that in *Olea europaea*, the hydroxylation of 7-deoxyloganic acid is not performed by a CYP72 (as it is in *Catharanthus roseus*), but rather by an oxoglutarate-dependent dioxygenase (*7eLAS*). Therefore, the upregulation of this CYP72 within our verbascoside QTL suggests it may instead possess a distinct, verbascoside-specific oxygenase activity or an alternative role in the phenylpropanoid shunt, rather than functioning as a classical iridoid 7DLH. Moreover, based on the recent published studies of the verbascoside pathway in other Lamiales species like *Ligustrum robustum* (a member of the Oleaceae family, like the olive) and *Cistanche tubulosa* [15, 20], this study successfully identified putative olive homologs for nearly all essential enzymatic steps. KEGG pathway enrichment analysis pinpointed genes encoding key enzymes such as TAL, 4-coumarate-CoA ligase (4CL), BAHD acyltransferases (SHCTs), and, remarkably, the final CYP hydroxylase required to complete the synthesis. The *OeACy3G* gene is a known rhamnosyltransferase, responsible for converting osmanthuside A into B, which is further hydroxylated to form verbascoside in *L. robustum* [15] and is also a homolog gene in *C. tubulosa,* expressed at T4/T5 to ensure the continuous supply of the osmanthuside B precursor. The expression patterns for nine candidate CYP450s were correlated with verbascoside accumulation. The dPCR analysis strongly validates that the transcriptional regulation of CYP genes is the rate-limiting factor for verbascoside synthesis. Although our dPCR-validated CYP genes map elsewhere, the Chr21 QTL contains 35 transcription factors and zinc-finger proteins among its 193 genes, providing a compelling trans-acting mechanism where this major locus remotely regulates downstream verbascoside synthesis. The four selected CYP identified as the main candidate genes belong to the subfamily CYP71D55 which are homologs of CYP98A in *C. tubulosa,* one of the main enzymes in verbascoside synthesis [20]. These genes show peak expression at the T5 stage (165 DAF), perfectly coinciding with maximum product accumulation in high-verbascoside cultivars [16–18]. Conversely, their near-zero expression in low-verbascoside cultivars at T5 confirms their essential role together with the cultivar-specific expression.

While our transcriptomic and dPCR analyses provide a temporal map across five developmental stages (from 45 to 165 DAF), the phenotypic quantification of verbascoside and the subsequent classification of cultivars into ‘high’ and ‘low’ groups were based solely on the 165 DAF time point. This specific stage was pre-determined as the peak accumulation period for verbascoside in olive drupes. During late fruit maturation, the degradation of oleuropein provides the necessary precursors (e.g. hydroxytyrosol) for verbascoside synthesis, leading to its maximum accumulation, a trend extensively documented across various olive cultivars [16–18, 37]. Selecting this peak accumulation phase ensured that we captured the maximum phenotypic divergence required to robustly contrast the extreme groups. The identification of candidate genes (CYPs and BAHD acyltransferases) and a major QTL on Chr21 controlling verbascoside accumulation are significantly strengthened and contextualized by the recent discovery of key enzymes in the competing secoiridoid pathway in *Olea europaea* [38]. The verbascoside molecule requires hydroxytyrosol (derived from the secoiridoid degradation pathway or a separate Tyr/Phe pathway) and a caffeoyl-glucosyl rhamnose moiety to be synthetized. As Calderini et al. [38] demonstrated, the genes encoding key oleuropein-pathway enzymes (such as *7eLAS, 7eLAMT*, and *OS*) are transcriptionally down-regulated during fruit ripening. Our data perfectly complement this by showing a concurrent peak in the expression of verbascoside-specific assembly enzymes (*OeCYP71D55* and *OeACy3G*) at 165 DAF, confirming a coordinated metabolic shunt where precursors are redirected from oleuropein to verbascoside biosynthesis during late maturation.

While allelic variation can significantly alter metabolic output by affecting overall enzyme efficiency, as observed in other fruit crops [33, 35], our analysis of variation from WGS data revealed a relatively low number of variants with a predicted functional impact, suggesting that the core enzymes of the verbascoside pathway are highly conserved. However, the identification of specific missense and nonsense mutations in core pathway genes, such as shikimate O-hydroxycinnamoyltransferase-like, anthocyanidin 3-O-glucoside 6″-O-acyltransferase, and cytochrome P450 71A6-like in a high-producing cultivar strongly suggests that the functional efficacy of specific enzyme isoforms dictates the difference between high- and low-verbascoside accumulating cultivars.

## Conclusion

This research provides a comprehensive and compelling dissection of the genetic basis of verbascoside biosynthesis, combining metabolomics, QTL mapping with transcriptomics and variant analysis, the study successfully identified a major genetic locus and a suite of high-confidence candidate genes responsible for the diversity of verbascoside content in olive. The findings have immediate scientific implications for further functional studies as well as in the cultivar selection for next olive breeding programs. Given the high expression levels of the newly identified genes, they could be readily converted into molecular markers for marker-assisted selection (MAS) and therefore validated. This would enable breeders to screen seedlings for high-verbascoside potential, significantly accelerating the development of new cultivars with enhanced nutritional and health-promoting properties. Observing and correlating the verbascoside accumulation trends at earlier developmental stages (e.g. 105 or 135 DAF) would have provided a continuous phenotypic trajectory to parallel our transcriptomic profiles. Such an approach would significantly strengthen the reliability of the high/low grouping and the generalizability of our conclusions. Future integrative studies should aim to couple multi-time-point metabolomic profiling with transcriptomics to further dissect the temporal dynamics of this pathway. The next step will be the functional validation of the top candidate genes. In the near future by the use of techniques such as heterologous expressions in *E. coli* or yeast, or gene editing via CRISPR/Cas9 in olive protoplasts, it will be possible to confirm the enzymatic function of the identified CYPs and acyltransferases.

## Materials and methods

### Analysis of phenolic compounds of fruit content in F1 progeny

Olive fruits were collected in two consecutive years, from a full-sib F1 progeny—represented by 94 15-year-old trees—and their parental varieties, Leccino and Dolce Agogia, field planted at CNR –Institute of Biosciences and Bioresources (Perugia, Italy). The harvest was carried out in early October, when most fruits were at a ripening index of 3 [39]. For the F1 progeny, as is standard in tree breeding where replicating 15-year-old trees is not feasible, the replicates refer to three technical extractions from a pooled sample of fruits collected from the single unique genotype. Fruits were randomly chosen around the canopy, promptly frozen in liquid nitrogen, and stored at −80°C.

Phenols were extracted from olive fruits, following the protocol described previously [5] with minor modifications. Frozen olive fruits stored at −80°C (1.5 g) were mixed with 6 ml of dimethyl sulfoxide (DMSO) and homogenized with a mortar and pestle. The mixture was then sonicated for 5 min, vortexed for 1 min, and left for 24 hours at 4°C. Thereafter, the mixture was further sonicated for 5 min, vortexed for 1 min, and centrifuged for 10 min at 4000 g. The clear supernatant was filtered through a 0.22 μm PES (polyethersulfone) membrane filter and mixed with the internal standard (caffeic acid). The extraction was performed in a triplicate. Identification and quantification were carried out by liquid chromatography electrospray ionization ion trap mass spectrometry. Chromatographic conditions and the mass spectrometer parameters were fully described in Reference [40]. Identification of compounds was carried out by comparing retention times, m/z values, and fragmentation spectra with those of authentic standards in negative ionization mode. Quantification was performed by building external curves dissolved in DMSO. The quantification of phenols was calculated by integrating the area under the peak measured from the extracted ion chromatograms (tolerance _0.5 Da).

### QTL analyses

For the Leccino × Dolce Agogia progeny, a genetic map was available, with a total of 16 743 segregating loci identified by ddRAD sequencing [23]. Data on phenolic compounds of fruit content of the mapped cross progeny have been used to perform a linkage analysis with the segregating mapped loci. QTL mapping was performed separately for each phenolic compound using the R/qtl package [41]. In detail, genotypic and phenotypic data were imported using the read.cross function and, to prevent issues related to markers with identical positions, slight random perturbations were introduced with the jittermap function. Conditional genotype probabilities were computed every 2.5 cM across the genome using calc.genoprob, assuming a genotyping error rate of 1%. A genome-wide scan was then conducted with the scanone function, applying Haley-Knott regression. LOD scores were calculated to identify genomic regions potentially associated with variation in phenol content. To define QTL regions, we applied the commonly used LOD threshold of >3, which was comparable to the 5% genome-wide significance threshold of 3.39 obtained from 1000 permutation tests. In order to map the QTL regions onto the public genome of the cultivar Arbequina [42] and identify all genes within these regions, the CDS sequences of Arbequina were aligned to Leccino draft genome (OLGENOME, http://olgenome.crea.gov.it/) already anchored to the Leccino and Dolce Agogia maps [23] using BLASTn [43] with default parameters. The position of the genes located within the QTL regions identified in the genetic maps was then used to define the corresponding QTL regions in the Arbequina reference genome.

### Metabolomics and transcriptomics analyses

Plant material: based on previous information on the phenolic profile, seven distinct cultivars were specifically chosen as they represent a high level of variation (contrasting high vs low groups) in fruit phenolics content: Coratina (COR), Dritta di Moscufo (DRI), Leccino (LEC), Tendellone (TEN), Leccio del Corno (LDC), Ascolana Tenera (AT), and Shengeh (SHE) [5, 37]. For the cultivars, three biological replicates collected from three distinct trees per cultivar grown in the same experimental field. The specific fruit phenolics content data confirming these variations at 165 DAF is provided in Fig. 3. Fruits used for RNA-Seq analyses were harvested from 45 to 165 days after full bloom (DAF) every 30 days, while the analysis of phenolic composition was performed on the same fruits (used for RNA-Seq), collected at 165 DAF from plants of an olive cultivar collection at the experimental field of the CNR-IBBR (Boneggio, Perugia) in central Italy. Plants were grown under the same environmental and agronomical conditions. Immediately after harvesting, the olive fruits were frozen in liquid nitrogen and stored at −80°C until further analysis. The phenols extraction of cultivars has been performed with the same procedure of progeny.

### RNA extraction, sequencing and processing of raw data

Total RNA was extracted from pulp of seven cultivars in five different time points using the RNeasy Plant Mini Kit (Qiagen), according to manufacturer’s instructions. To avoid DNA contamination, each sample was treated with DNase I (Ambion). Library preparation and sequencing of the 35 samples (7 cultivars × 5 time points) were outsourced to Macrogen Europe (Amsterdam, Netherlands). Libraries were prepared using the TruSeq Stranded mRNA LT Sample Prep Kit (Illumina) and sequenced on an Illumina platform in paired-end mode (2 × 150 bp). Adapter sequences and low-quality bases at the 3′ end of short reads were removed using CUTADAPT [44] and ERNE-FILTER [45], respectively. After trimming, only read pairs in which both reads exceeded 50 bp were retained. The filtered reads from each sample were then aligned to the *Olea europaea* cv. Arbequina reference genome [42] using STAR v2.7.9a [46] with default parameters. The htseq-count Python utility [47] was used to calculate gene-level read counts, considering only uniquely mapped reads. The resulting counts data were used in the following differential gene expression (DE) analysis in R environment.

### Differential gene expression analyses

Based on the terminal phenol content at 165 DAF, the cultivars were grouped into three categories: Low, Medium, and High for each of the nine phenol types. Transcriptomic contrasts between the ‘High’ and ‘Low’ groups were then performed separately at each of the five developmental time points (T1–T5). To conduct the analysis, cultivars within the ‘High’ category and ‘Low’ category were pooled to form two distinct conditions. The contrasts were made directly between the pooled ‘High’ and ‘Low’ groups using DESeq2, treating the different cultivars within each category as biological replicates for that specific phenotype. This pairwise comparison was repeated for each of the nine phenol types across each time point (T1–T5). Raw counts data were used as input for the DE analysis with the Bioconductor package DESeq2 [48] applying the VST, the Wald statistic test, and correcting for multiple testing with the Benjamini-Hochberg false discovery rate (FDR) method. Expression heatmaps for each DE analysis were made with the pheatmap v.1.0.12 R package. The Phenogram software (https://visualization.ritchielab.org/phenograms/plot) was utilized for visualizing chromosomes and DEGs’ position relative to the QTL regions. The relationships among the DEG sets, e.g. number of shared genes and unique genes, were achieved by means of the UpSetR v.1.4.0 R package.

### Functional enrichment analysis of DEGs

Genes found to be significant DE at least at one time point were used for enrichment analysis by means of the clusterProfiler v4.10.1 R package [49] which allowed Gene Set Enrichment Analysis (GSEA) and over-representation analysis (ORA) in GO and KEGG databases. The maximum of the T1–T5 |log2fc| values was assumed to be unique log2fc for each DEG, so that down-regulated and up-regulated genes could be distinct depending on whether log2fc < −1 or log2fc > 1.

### Polymorphisms and structural variants in olive WGS dataset

The genetic variation of thirteen olive cultivars was analyzed using WGS data obtained from the NCBI repository. The specific accessions are as follows: Arbequina SRR9860530; Chemlal-de-Kabylie SRR9860531; Frantoio SRR9860541; Grappolo SRR9860495; Hojiblanca SRR9860536; Kalamon SRR9860537; Konservolia SRR17024196; Koroneiki SRR9860523; Leccino SRR9860534; Manzanilla-de-Sevilla SRR9860524; Morrut SRR9860540; Picual ERS4046989; and Picudo SRR9860519. The selection was based on the quantity of verbascoside in these cultivars from the author’s previous publication [5]. The cultivars were divided based on their high, medium, and low phenol content, and their reads were aligned on the Arbequina assembly using BWA-MEM2 v2.2.1. Subsequently, SNP and INDELs joint calling was performed using freeBayes v1.3.6 and only variants labeled ‘PASS’ and supported at least 6 reads were retained. Functional annotation of variants was achieved with SnpEff v5.2c [50], while GOterms and protein descriptions were retrieved from the Arbequina general feature format (GFF) file and incorporated into the variant Call Format (VCF) using variant effect predictor (VEP) v111 [51]. Structural variations (SVs) were detected by means of DELLY v1.2.6 [52] and annotations of genes affected by SV or putative gene fusions were performed with sansa v0.2.1. Three subsets of variants were made extracting those included in coding sequences, grouped as (SNPs and INDELs <100 bp), (deletions >100 bp), (insertions, inversions and duplications >100 bp). A circos plot representation was made with pyCirclize v1.10.0 (https://github.com/moshi4/pyCirclize), which displays, besides the three variant subsets, the genomic regions containing DEGs, and another track, showing all genes associated with verbascoside synthesis. For better clarity, the genome was represented on a binned scale with fixed bins of 2.265 Mb, and variants of densities (e.g. deletions-containing bases divided by bin size etc.) were computed inside each bin.

### Digital PCR validation of candidate genes

All reactions were run on a 96-well platform (QIAcuity Nanoplate 8.5 k 96-well, QIAGEN, Hilden, Germany). For absolute gene quantification, reactions were performed in a total volume of 12 μl with 15 ng of cDNA, 4 μl from 3× EvaGreen PCR Master Mix and 0.5 μM of each primer pair.

Candidate genes were verified using RNA isolated from four cultivars (Ascolana Tenera and Coratina, which had high verbascoside content; together with Tendellone and Shengeh, which had low verbascoside content). The same fruits collected for RNA-Seq experiment have been used for genes validation. Primers were designed using the program Primer3 version 4.1.0 (Table S24). Relative quantification by dPCR was based on the absolute concentration of the target genes in the original, undiluted sample, which was then normalized using the reference gene (EF1α) [53]. Reactions were performed on three biological cDNA replicates for each sample.

### Statistical analysis of metabolomics and genes validation data

Data were analyzed using Two-way ANalysis Of VAriance ()ANOVA) followed by Tukey’s multiple comparisons test of GraphPad Prism version 9.0.0 (GraphPad Software, San Diego, CA, USA, www.graphpad.com) (^****^  *P* = 0.000 and ^**^  *P* < 0.01), Tukey test was used to compare mean values. Box and whisker plots and histograms were made by the same software.

## Supplementary Material

Web_Material_uhag154

## Data Availability

RNA-Seq raw data has been deposited in the NCBI Sequence Read Archive (SRA) under the accession number PRJNA1329229. The identified genes have been deposited in the NCBI GenBank database under the following accession numbers: PX701745; PX701803; PX701802; PX701804; PX701805; PX702678; PX701806; PX701808; PX701807 and PX701809.
