# Supplementary material for: Unveiling the genetic blueprint of verbascoside biosynthesis in Olea europaea: an integrative metabolomics, genetics and transcriptomics approach
Source: Hortic Res. 2026 Apr 17;13(9):uhag154. doi: 10.1093/hr/uhag154 (PMC13518232; doi:10.1093/hr/uhag154)
Supplement: Supplementary file 1 [file web_material_uhag154.zip › Supplementary_Figures.pdf]

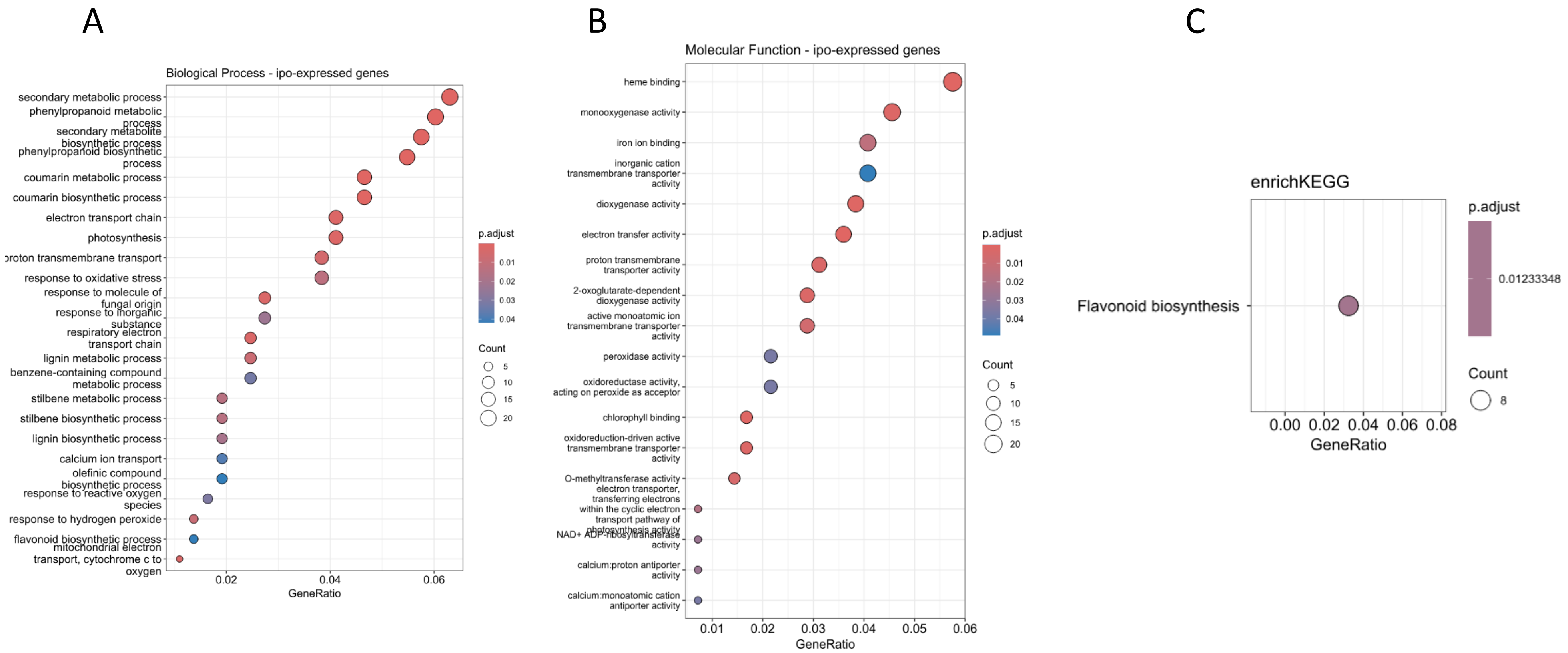

Figure S1. The enrichment analysis of tyrosol DEGs. Gene Ontology terms and KEGG pathway resulting from the subset of 679 lowly expressed genes (lfc<1) analyzed for over-representation by clusterProfiler; biological process (A), molecular function (B) GO categories and KEGG pathway (C) are shown. No significant enriched terms resulted from the highly expressed subset (480 genes).

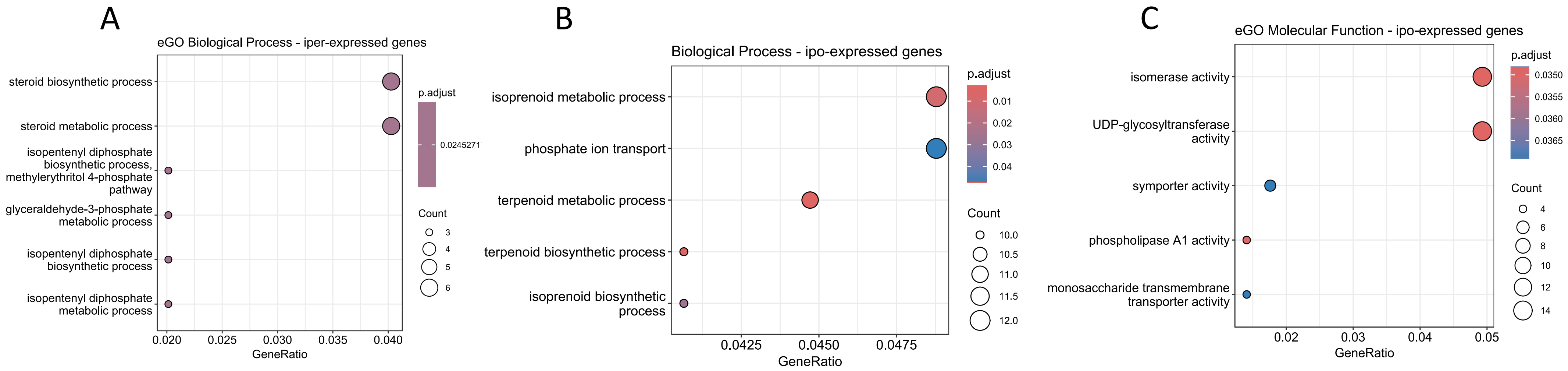

Figure S2. Hdroxytyrosol DEGs enrichment analysis. The highly expressed genes (n=484) over-representation analysis (A) detected 6 enriched terms in the biological process Gene Ontology category. The lowly expressed genes (n=294) enriched in 5 terms of the biological process (B) and 5 of the molecular function C) GO categories.

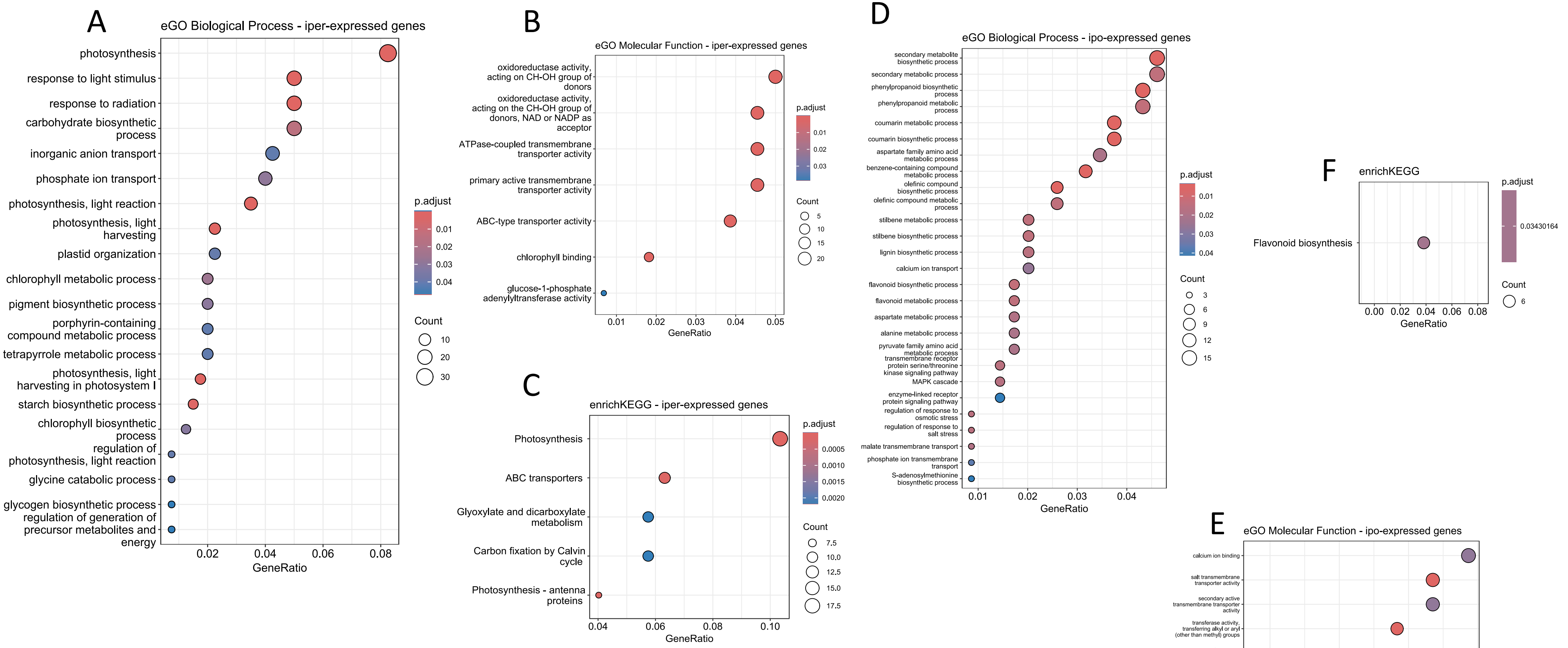

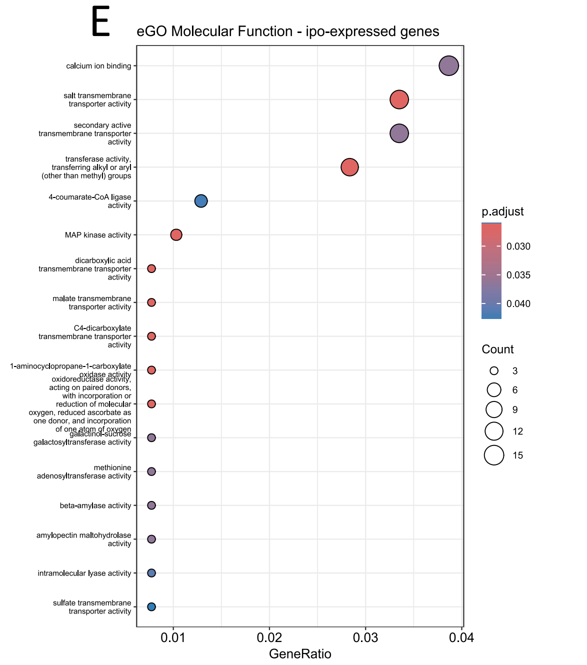

Figure S3. Oleuropein DEGs enrichment analysis. The subset of 711 highly expressed genes (lfc>1) over-representation analysis identified significant GO terms in the biological process (A), molecular function (B) categories and 5 KEGG pathways (C). DEGs with log2FC<1 (n=639) results are shown in (D, E, F) for the same analyses.

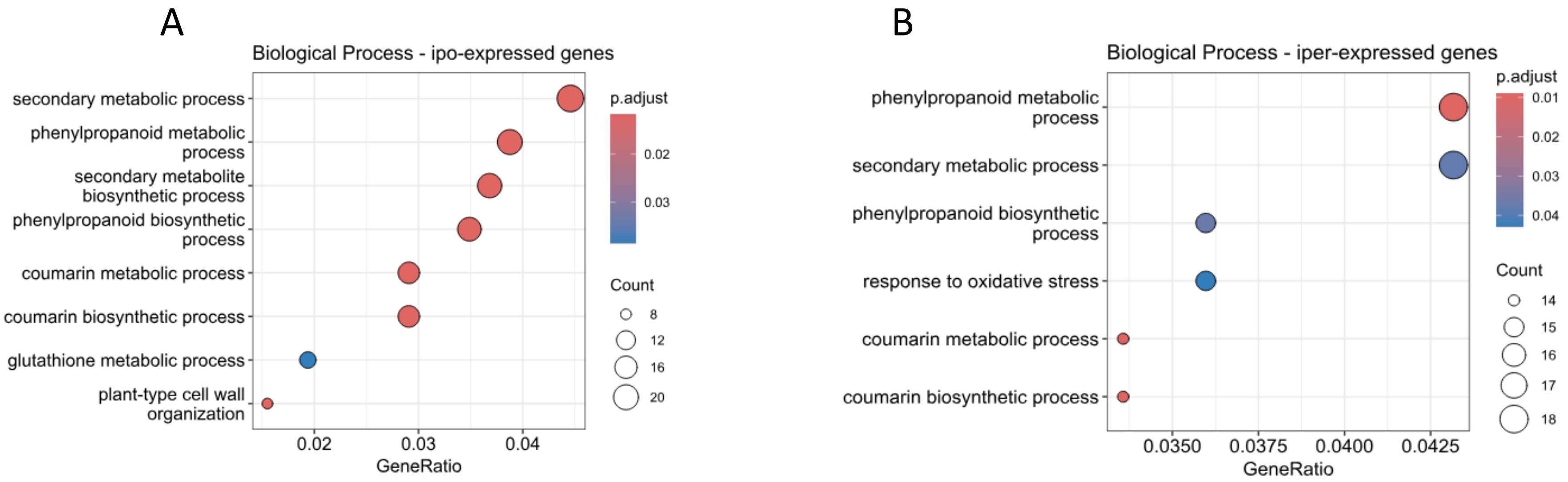

Figure S4. The Oleuropein aglycon DEGs over-representation analysis resulted in 8 terms for the higly expressed subset (n=888) (A) and 6 terms for the lowly expressed subset (n=988) (B) both in the biological process GO category.

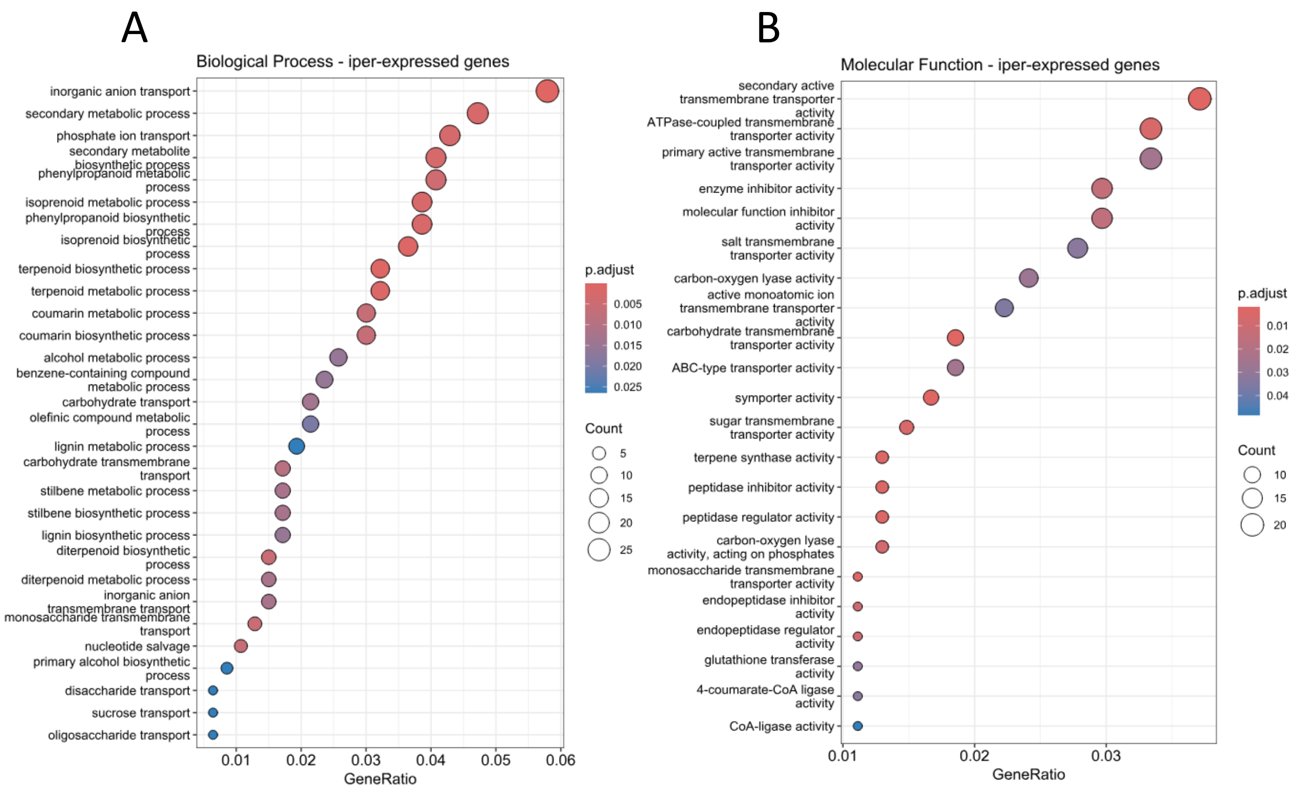

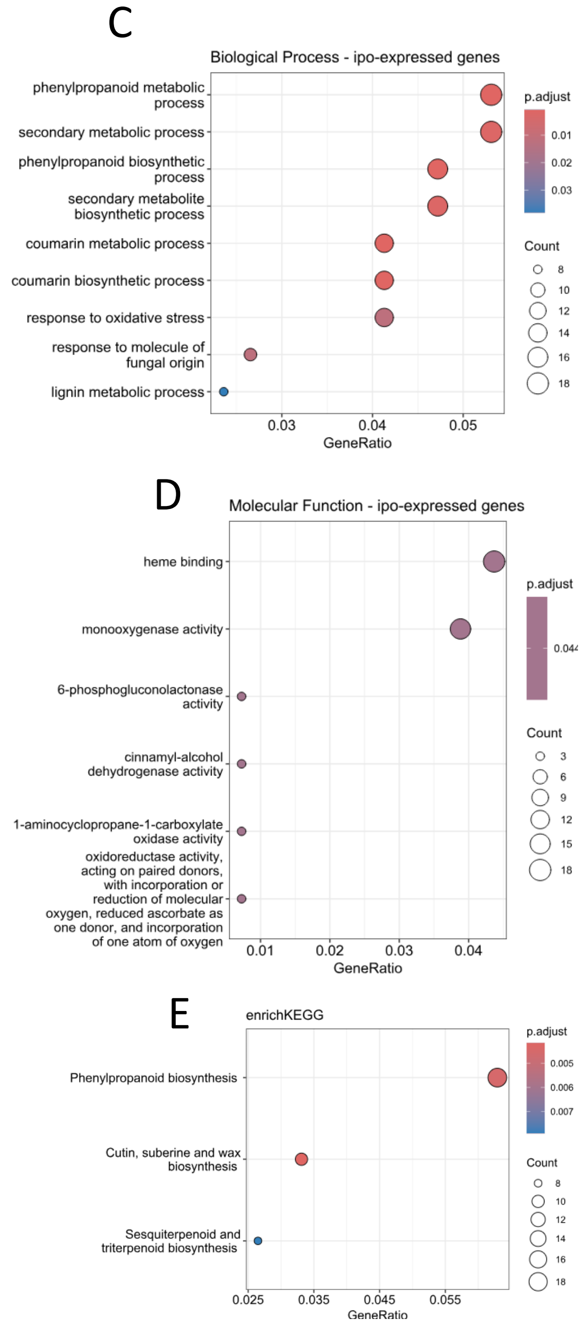

Figure S5. over-representation analysis of Ligstroside aglycon DEGs. Enriched terms of the biological process and molecular function GO categories yielded for both the highly expressed genes (n=908, A and B) and the lowly expressed genes (n=703, C and D). The KEGG over-representation analysis resulted in 3 significantly enriched terms, only for lowly expressed genes (E).

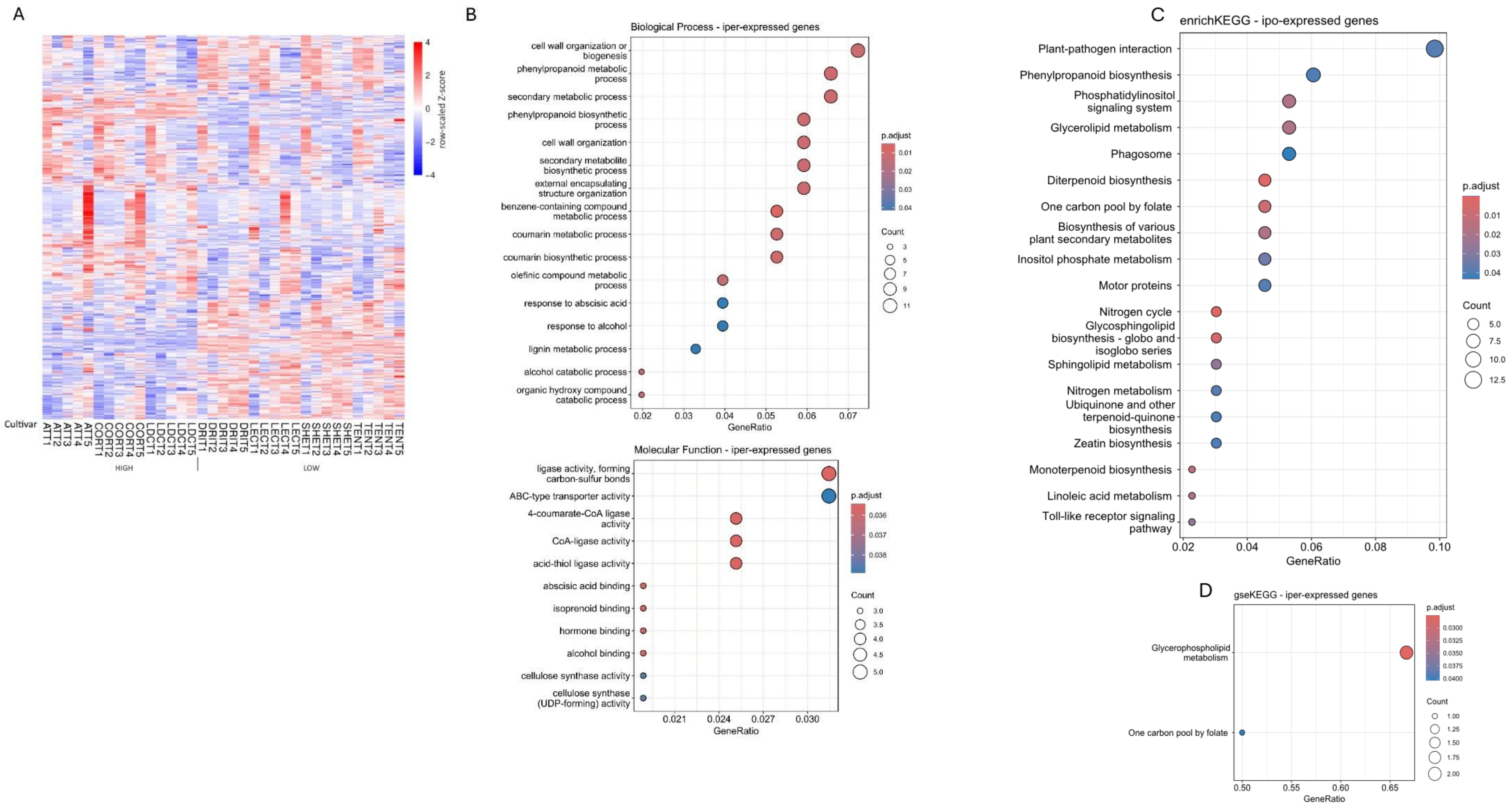

Figure S6. A) Heatmap with the variance stabilization transformation (VST, row-scaled Z-score) values of the 615 genes identified by pairwise differential expression analysis (adjusted p-value<0.05). Samples were grouped based on both phenotypes (Verbascoside levels: High: COR, AT, LDC; Low: DRI, TEN, SHE, LEC) and timepoints (T1-T5). B) Enrichment of up-regulated genes and their biological and molecular function. The analysis of verbascoside DEGs performed with the gseKEGG function of clusterProfiler, which use the Gene Set Enrichment Analysis method, identified 2 enriched KEGG pathways (C) for the highly expressed genes (n=266). Lowly expressed genes (n=236) were found to belong to the KEGG pathways shown in (D), identified by over-representation analysis.

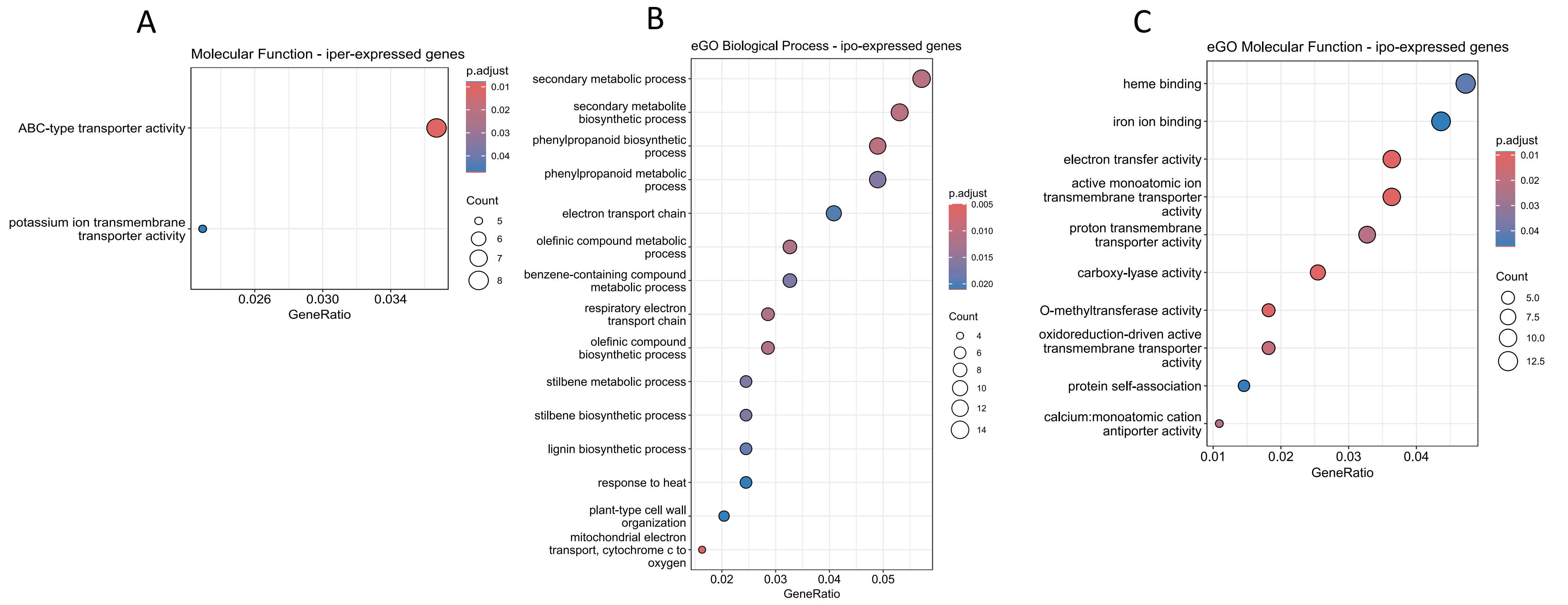

Figure S7. Over-representation analysis of apigenin-7-O-glucoside DEGs resulted in two molecular function terms (A) for the highly expressed gene subset (n=373), whereas the lowly expressed gene subset (n=436) is involved in both biological process (B) and molecular function (C) GO categories.

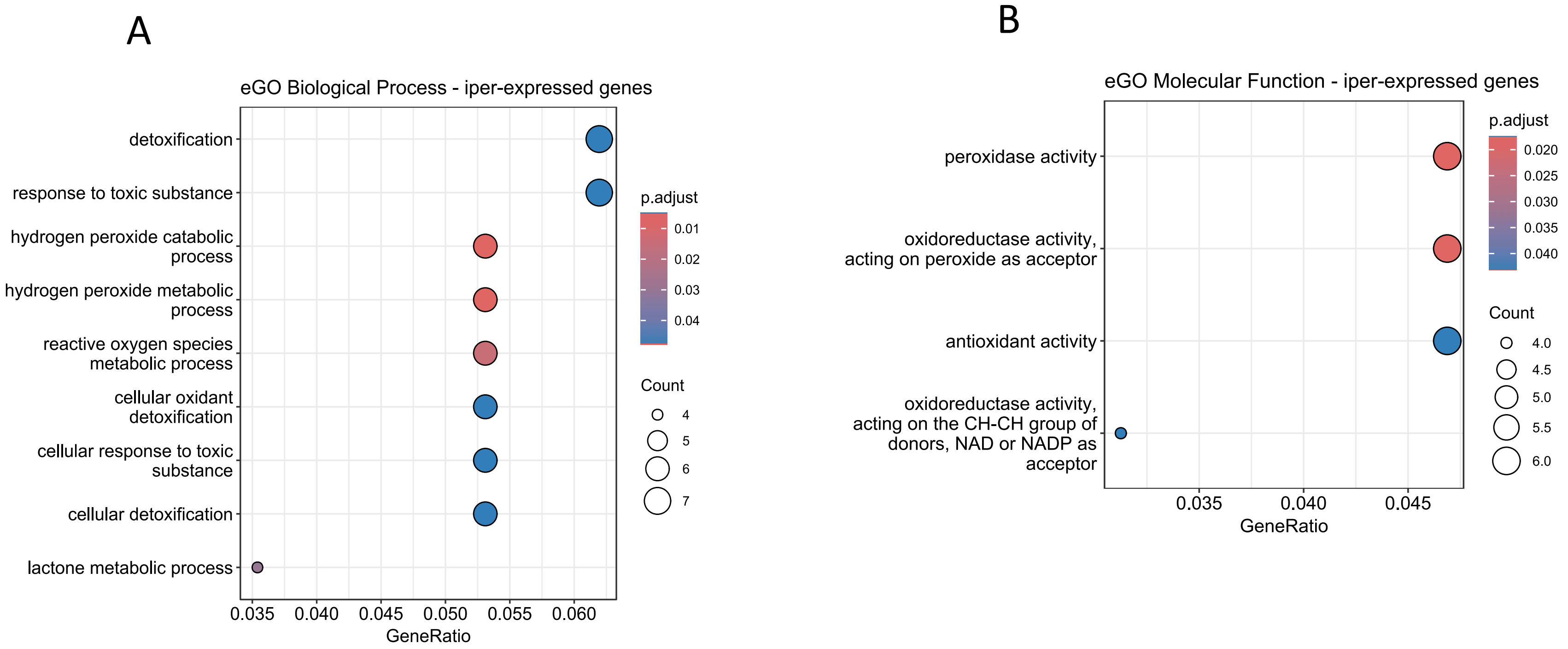

Figure S8. Results of the over-representation analysis performed on 215 highly expressed genes detected in the "HIGH" luteolin-7-O-glucoside samples group. The biological process GO category terms (A) and molecular function terms (B) are shown. No significant enriched terms were found for lowly expressed genes.

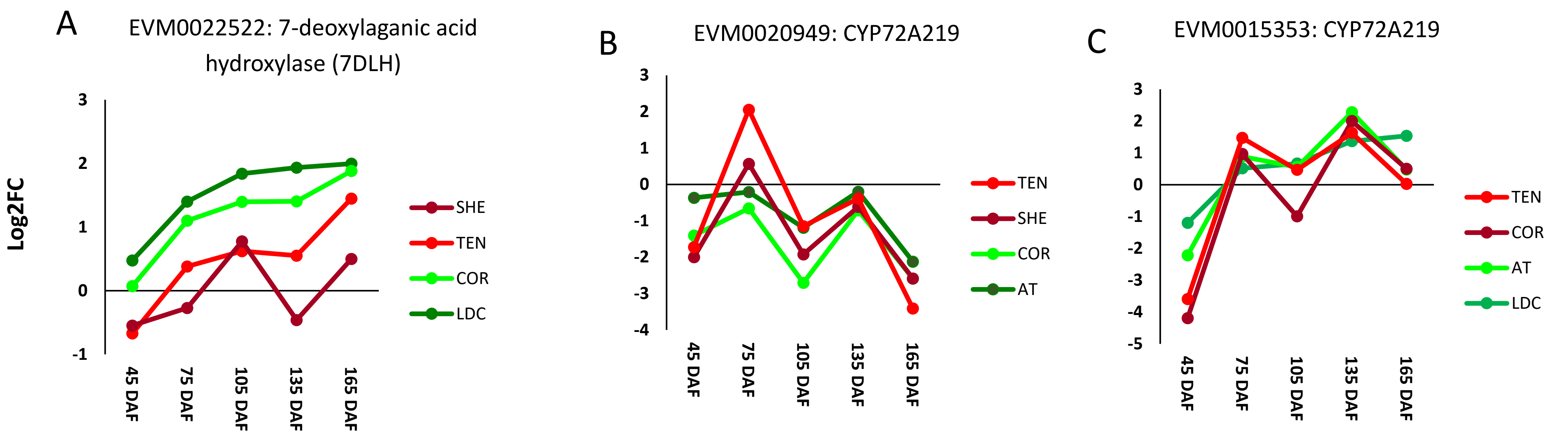

Figure S9. Expression of candidate genes, located in QTL regions and potentially involved in iridoid, verbascoside, and flavonoid biosynthesis, during fruit development. A) DE between cultivars with high (green) and low (red) content of oleuropein aglycon and ligostroside aglycon. B) Differential expression (DE) between cultivars with high (green) and low (red) verbascoside content. C) DE between cultivars with high (green) and low (red) rutin content.

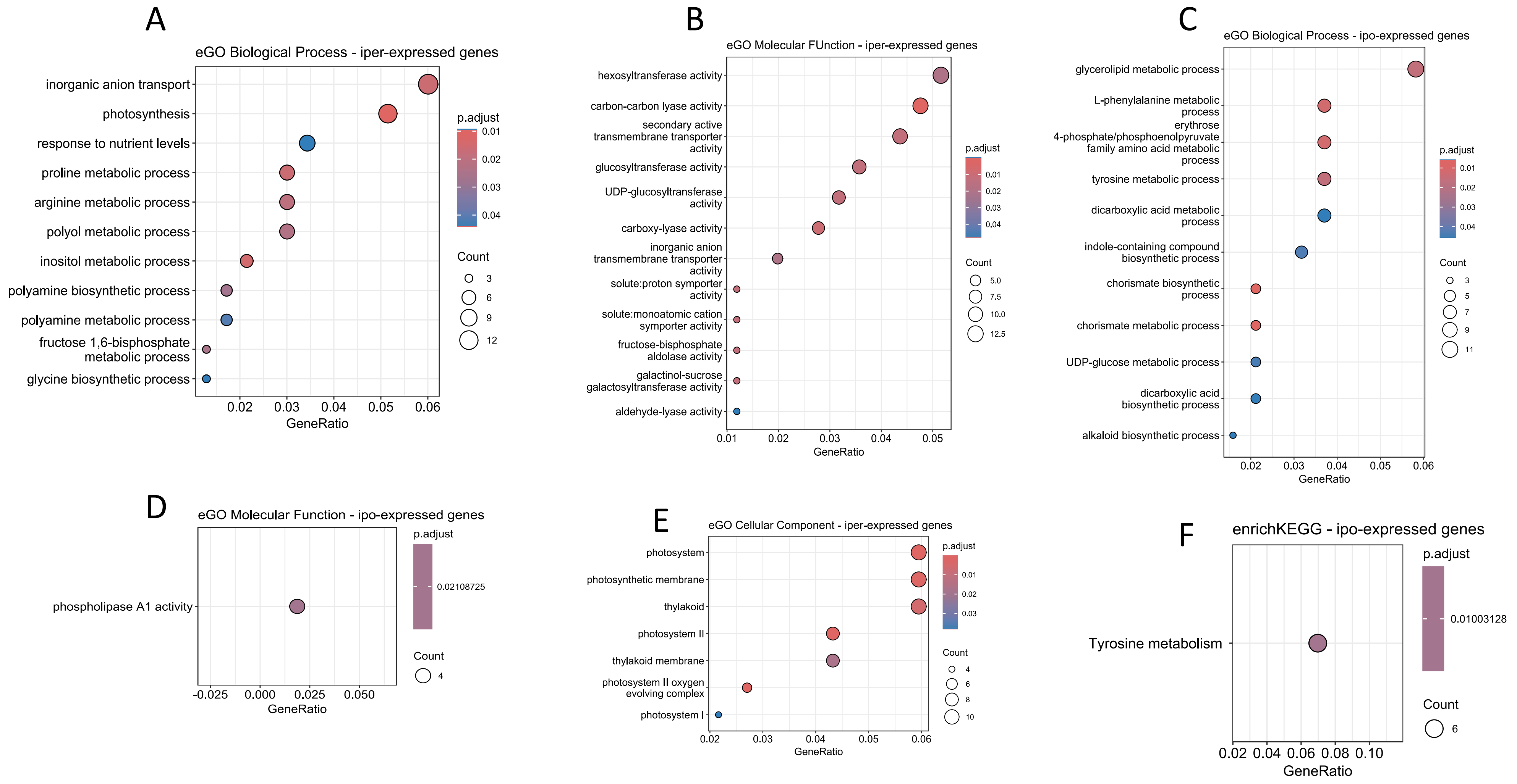

Figure S10. Over-representation analysis of the Rutin sample group DEGs. Enriched terms of the biological process and molecular function GO categories were yielded for both the highly expressed genes (n=410, A and B) and the lowly expressed genes (n=352, C and D). In addition, significantly enriched terms of the GO cellular component category for the highly expressed genes (C) and one KEGG pathway for the lowly expressed genes (F) were found.

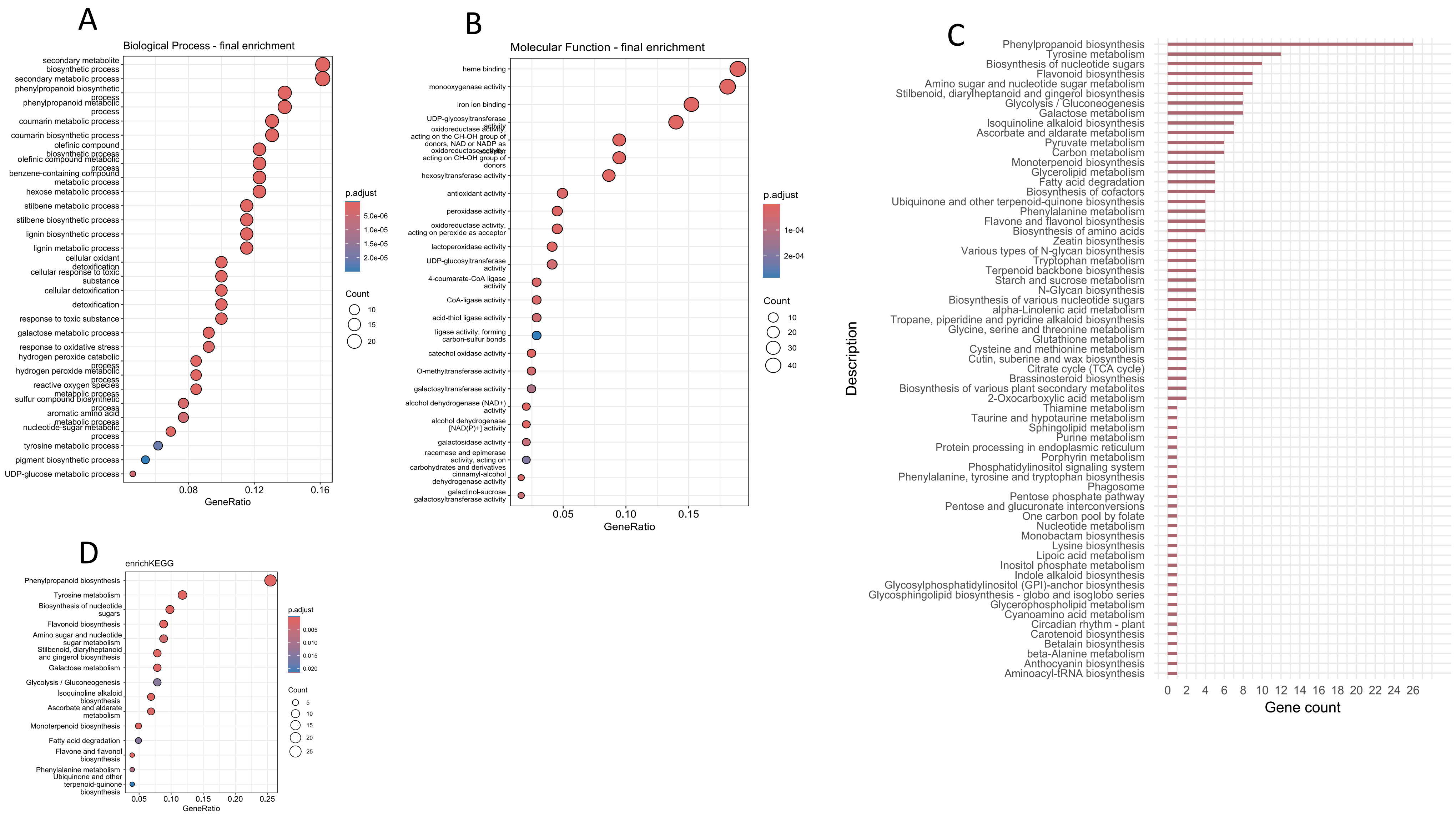

Figure S11. Results of a final enrichment analysis on 301 genes identified in this study, which leveraged an integrated analysis of QTL, transcriptomics, and sequence homology data. The processes shown here in bubble/bar plots, that is GO biological processes (A), molecular functions (B), and KEGG pathways (C, D) confirm the involvement of these genes in most of the expected pathways of the iridoid metabolic network, and specifically in verbascoside synthesis.

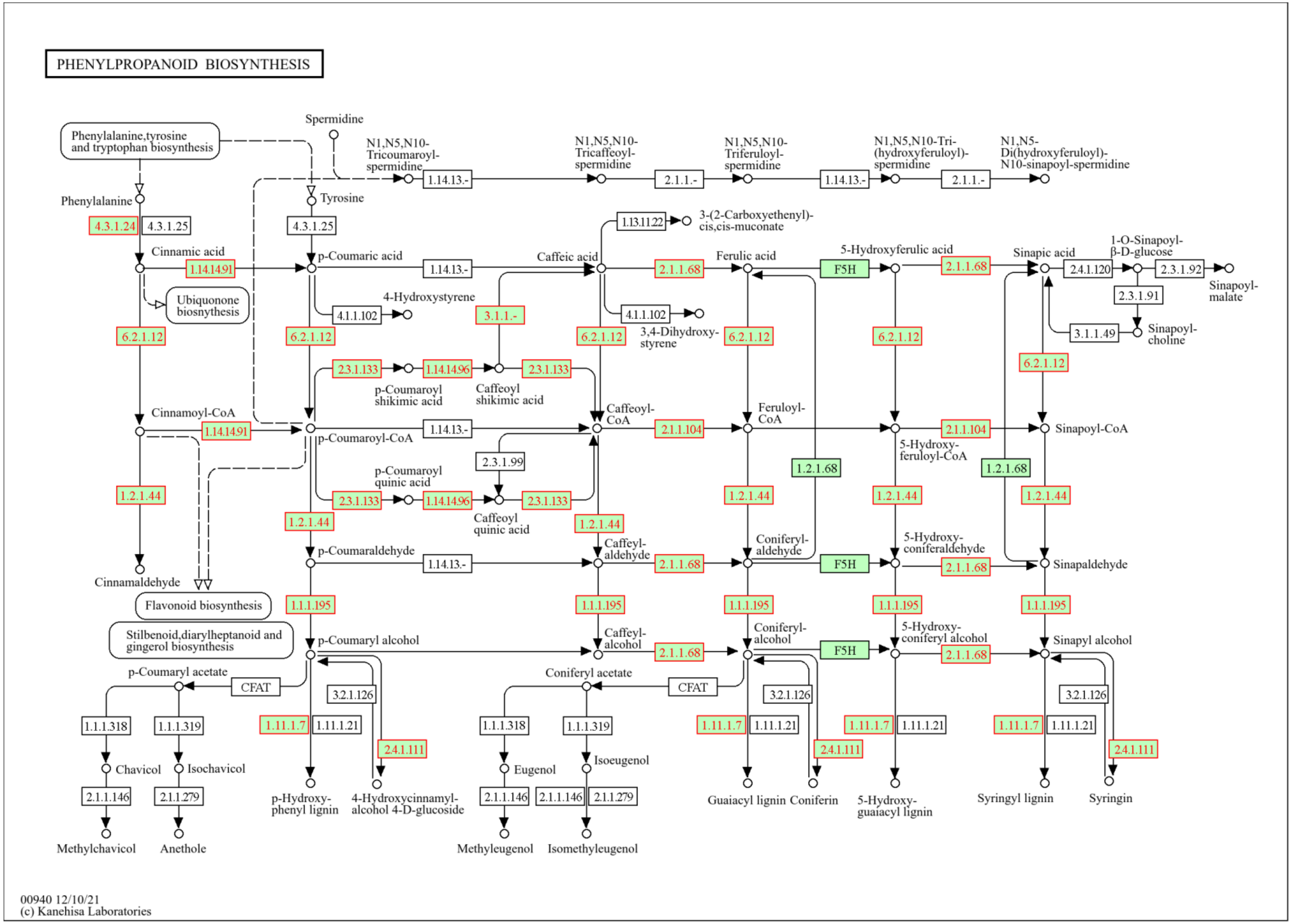

Figure S12. KEGG diagram for the phenylpropanoid pathway in *O. europaea* v. sylvestris (https://www.kegg.jp/pathway/oeu00940). The 26 enzymes identified belong to the enzyme classes in red. The gene IDs were converted from Arbequina to Sylvestris: 111399405, 111396236, 111403111, 111411999 111388752, 111380387 111372687, 111387805 111368470, 111412792 111397542, 111386908 111391169, 111383563 111385307, 111367091 111375374, 111367232 111389230, 111375382 111390675, 111367736 111404591, 111375693 111375802, 111388454.

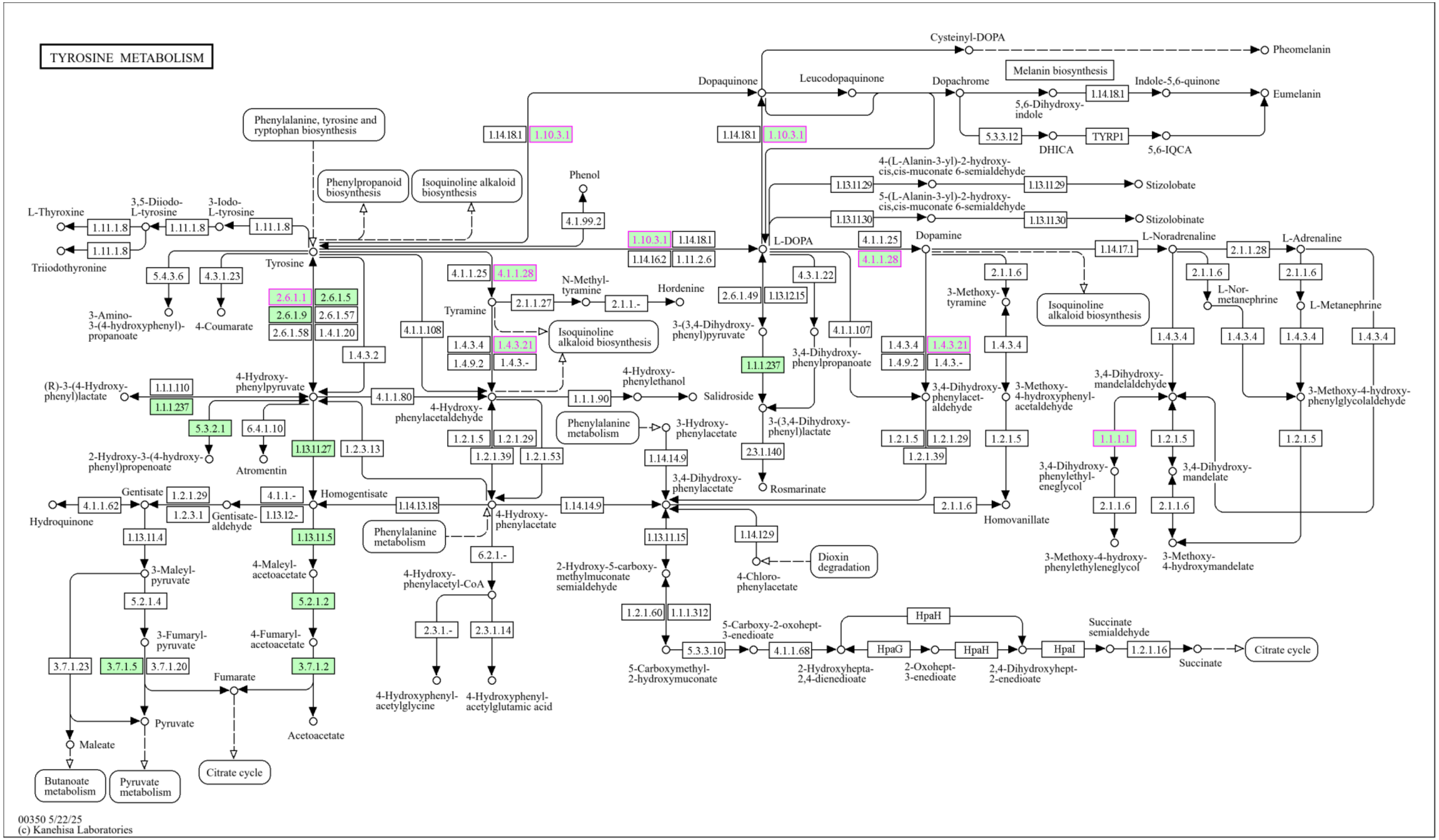

Figure S13. KEGG diagram for the tyrosine synthesis pathway in O. europaea v. sylvestris (https://www.kegg.jp/pathway/oeu00350). The 12 enzymes whose genes were found among either in our set of DEGs or in QTL regions belong to the enzyme classes in purple. The 12 Sylvestris gene IDs are: 111370179, 111370494, 111370324, 111370180, 111390067, 111373593, 111372580, 111374154, 111399970, 111397554, 111374857, 111384383.

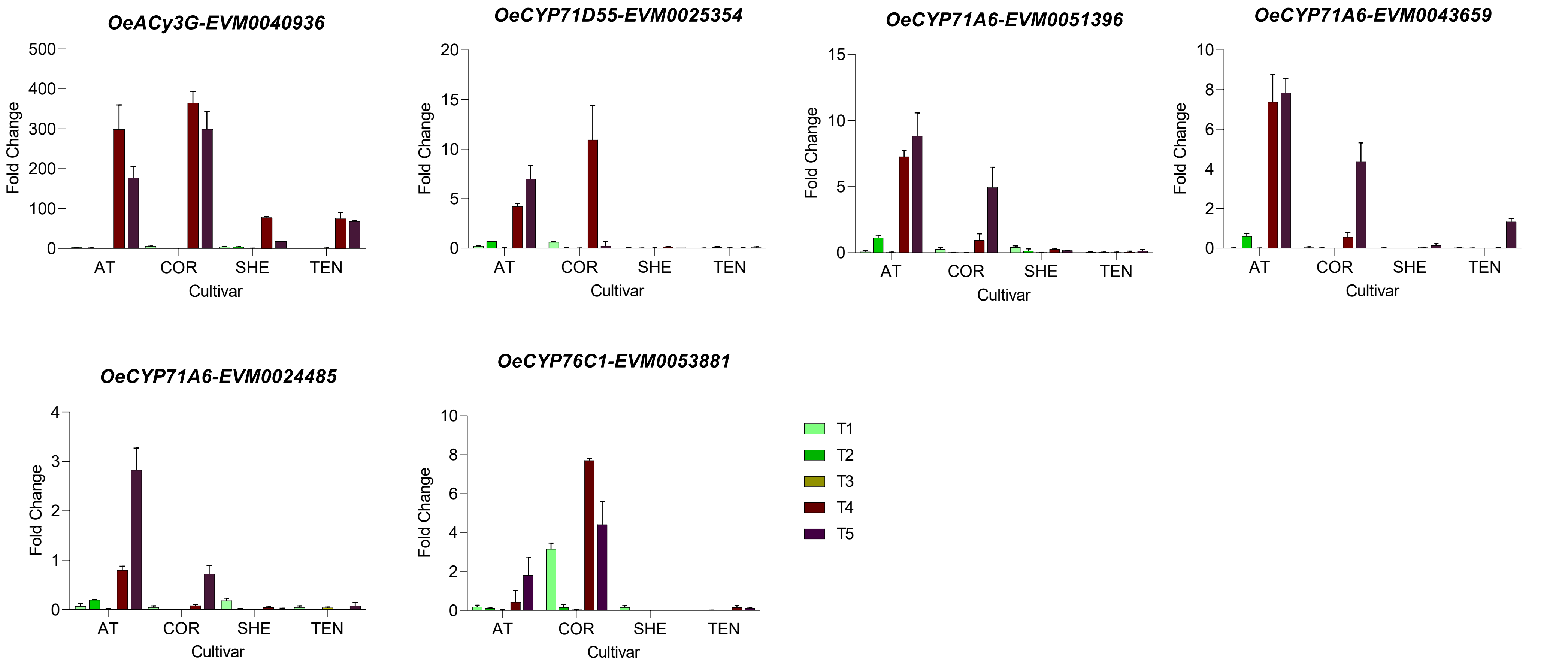

Figure S14. Relative quantification by dPCR of the mRNA expression of six genes based on the absolute concentration of the target genes which was then normalized using the reference gene (*EF1α*). Tukey's multiple comparisons test results show significant differences (**** $p < 0.0001$) among time point in each cultivar and between cultivars for the same genes.
